# SNRPC promotes chemoresistance in Wilms tumor via the NF-κB-CXCL17 axis regulating M2-Type TAMs infiltration and targeted nanotherapy research

**DOI:** 10.1186/s13046-026-03680-z

**Published:** 2026-02-28

**Authors:** Xiangpan Kong, Li Lei, Liming Jin, Chunnian Ren, Tao Mi, Quan Wang, Dawei He

**Affiliations:** 1 Department of Urology, Children’s Hospital of Chongqing Medical University, National Clinical Research Center for Child Health and Disorders, Ministry of Education Key Laboratory of Child Development and Disorders, Children Urogenital Development and Tissue Engineering of Chongqing Education Commission of China, The Laboratory of Targeted Delivery of Traditional Chinese Medicine, Chongqing, 400014 P. R. China; 2 Department of Cardiothoracic Surgery, Children’s Hospital of Chongqing Medical University, Chongqing, P. R. China; 3 The Second Affiliated Hospital of Chongqing Medical University, Key Laboratory of Integrated Therapy of Traditional Chinese Medicine for Tumors, Chongqing Municipal Administration of Traditional Chinese Medicine, Chongqing, P.R. China

**Keywords:** Wilms tumor, Chemoresistance, M2-type tumor-associated macrophages, SNRPC, NF-κB, CXCL17, Nanodrug delivery, Migrasomes

## Abstract

**Background:**

Wilms tumor (WT), the most common pediatric malignant renal tumor, shows high recurrence in high-risk subtypes due to chemoresistance. Tumor microenvironment (TME) remodeling, particularly M2-type tumor-associated macrophages (TAMs), contributes to chemoresistance, but underlying mechanisms remain unclear. This study explored TME-related chemoresistance mechanisms in WT and developed targeted therapeutic strategies.

**Methods:**

Clinical WT samples were analyzed for M2-type TAMs infiltration and SNRPC expression. Bioinformatics analysis of TARGET-WT data identified M2-associated genes. In vitro experiments (cell transfection, qRT-PCR, Western blot, co-culture, ChIP and dual-luciferase reporter assays) explored SNRPC’s role in regulating M2-type TAMs. Animal models (orthotopic tumor and lung metastasis) verified in vivo effects. A hybrid exosome nanosystem (DOX/siSNRPC@hEVs) was constructed and evaluated for efficacy and safety. Statistical analyses included t-test, ANOVA, and survival analysis.

**Results:**

M2-type TAMs (CD68⁺CD163⁺) infiltration was higher in chemoresistant WT and associated with poor prognosis. SNRPC was overexpressed in chemoresistant WT, correlated with M2-type TAMs, and promoted tumor malignancy and M2-type TAMs polarization. Mechanistically, SNRPC activated NF-κB signaling, inducing CXCL17 upregulation to recruit M2-type TAMs, with partial CXCL17 release via migrasomes. DOX/siSNRPC@hEVs showed high targeting, reduced toxicity, inhibited tumor growth/metastasis, and reversed chemoresistance by reducing M2-type TAMs.

**Conclusions:**

The SNRPC-NF-κB-CXCL17-M2 TAMs axis drives WT chemoresistance. DOX/siSNRPC@hEVs effectively targets this axis, providing a novel strategy for high-risk WT.

**Graphical abstract:**

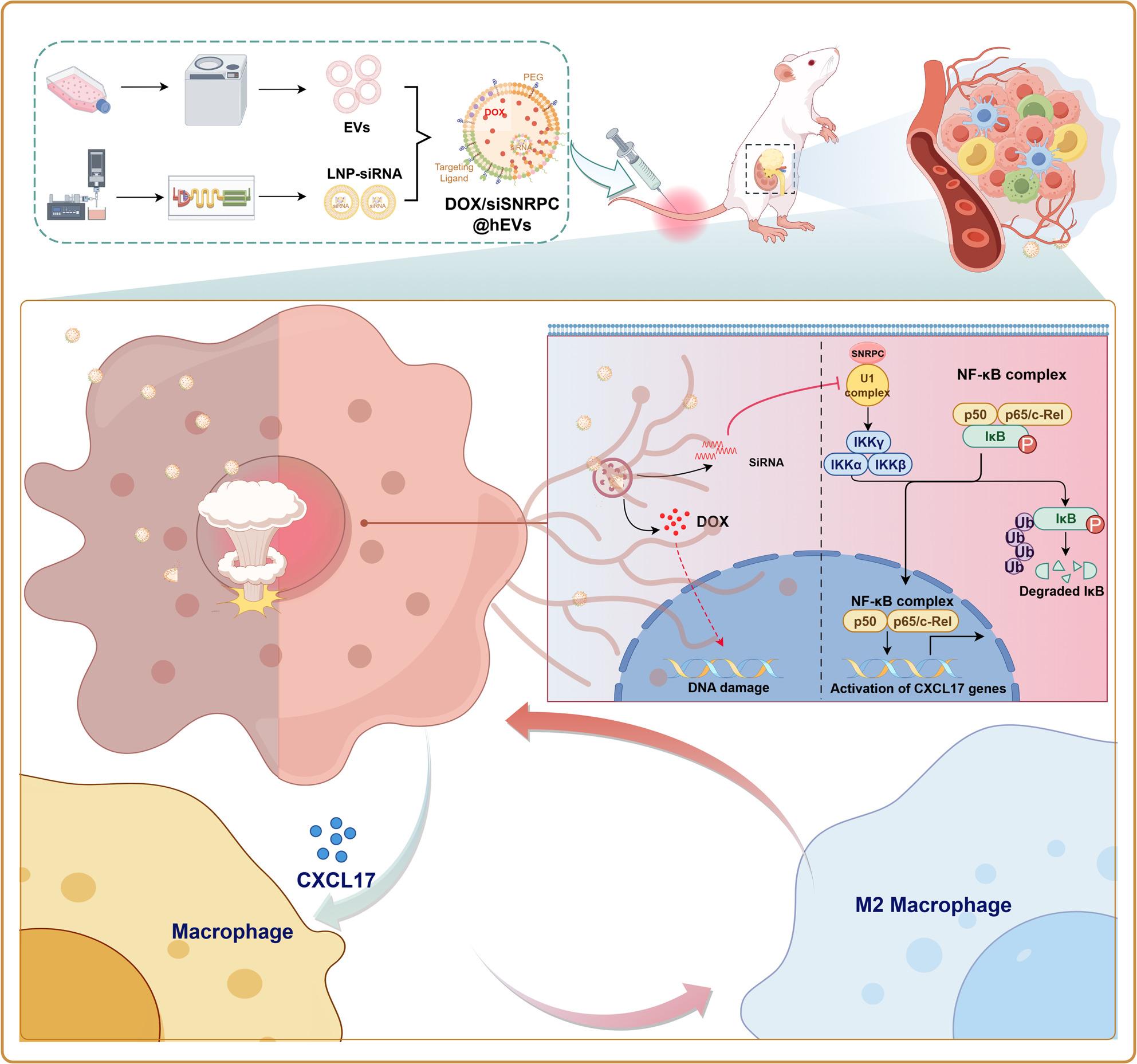

**Supplementary Information:**

The online version contains supplementary material available at 10.1186/s13046-026-03680-z.

## Introduction

 Wilms tumor (WT), the most common pediatric malignant renal tumor, is primarily treated with a combination of surgery, chemotherapy, and radiotherapy [1]. While this multimodal approach has achieved curative effects in most patients, the recurrence rate remains significantly high in high-risk subtypes, such as blastemal-predominant WT and diffuse anaplastic WT. Chemoresistance stands as the core obstacle leading to treatment failure [2, 3]. Studies have shown that approximately 50% of patients with anaplastic WT experience recurrence [4, 5]. Although conventional chemotherapeutic agents like doxorubicin are widely used in clinical practice, they are associated with severe side effects such as cardiotoxicity, highlighting an urgent need to explore new therapeutic targets and strategies [6–8].

Aberrant remodeling of the tumor microenvironment (TME) is a key driver of WT progression and chemoresistance [9, 10]. The complexity and dynamic plasticity of TME components directly influence treatment responses. The TME of WT consists of immune cells, stromal cells, extracellular matrix, and soluble factors, among which the infiltration pattern of immune cells is closely related to prognosis. A unique “immune desert” subtype exists in anaplastic WT, characterized by significantly reduced infiltration of immune effector cells such as CD8⁺ T cells and CD3⁺ T cells, accompanied by TP53 mutations and downregulation of the cGAS-STING innate immune pathway. This leads to tumor immune evasion and enhanced chemoresistance [11]. The formation of this immunosuppressive microenvironment involves multiple mechanisms, including abnormal expression of immune checkpoint molecules, dysregulation of cytokine networks, and metabolic imbalance.

Tumor-associated macrophages (TAMs), the most abundant immune cell population in the TME, play a crucial role in regulating WT progression and chemoresistance through their polarization status [12, 13]. M2-like TAMs promote an immunosuppressive phenotype by secreting anti-inflammatory factors such as IL-10 and TGF-β. Meanwhile, they remodel angiogenesis and stromal structure by releasing VEGF and CXCL family chemokines, providing a sanctuary for tumor cells [14]. Clinical studies have confirmed that the response to chemotherapy varies significantly among histological subtypes of bilateral WT (e.g., stromal-predominant subtype), which is essentially associated with the heterogeneity in TAM infiltration density, cytokine profiles, and degree of stromal fibrosis within the TME. This suggests that targeting TAM polarization may reverse treatment resistance [15]. Additionally, stromal cells such as fibroblasts and endothelial cells in the WT TME participate in maintaining the chemoresistant phenotype through crosstalk with tumor cells and immune cells, although their specific regulatory networks remain incompletely elucidated.

In terms of therapeutic strategies, studies on high-risk WT models have demonstrated that histone deacetylase inhibitors (e.g., panobinostat) can remodel the TME through epigenetic regulation, reduce M2-like TAM infiltration, and enhance the function of immune effector cells. Moreover, they show superior efficacy and safety compared to doxorubicin in patient-derived xenograft models (PDX) [5]. For patients with WT and extrapulmonary metastases, intensified chemotherapy regimens improve prognosis in some patients, but the 4-year event-free survival rate remains only 77.3%, underscoring the urgent need for TME-targeted therapies [16].

Nanoparticle-based delivery systems provide a novel tool for TME-targeted therapy [17, 18]. Exosome-liposome hybrid vesicles achieve efficient drug loading and targeted delivery through membrane fusion technology, enabling specific enrichment in the TME and modulation of the immune microenvironment [19]. In tumor models, they not only enhance the accumulation of chemotherapeutic agents like doxorubicin but also deliver siRNA to silence chemoresistance-related genes, laying the foundation for combination therapy [20, 21]. Similar studies have confirmed that pH/redox dual-responsive nanosystems co-delivering chemotherapeutic drugs and epigenetic modulators can reverse the tumor chemoresistant phenotype, suggesting the potential of combined strategies targeting both tumor cells and the TME [22].

In summary, deciphering TME-regulated chemoresistance mechanisms and developing targeted delivery systems are crucial for improving the prognosis of WT. Focusing on M2-like TAM-mediated chemoresistance, and based on the identified correlation between SNRPC(small nuclear ribonucleoprotein polypeptide C) and M2-like TAM marker genes, this study proposes that SNRPC may be involved in WT chemoresistance by regulating immune microenvironment-related signaling pathways. It also explores the application value of exosome-liposome hybrid systems in combination therapy for chemoresistant WT, aiming to provide experimental evidence for optimizing clinical treatment strategies.

## Materials and methods

### Clinical samples

Clinical specimens were collected from patients diagnosed with Wilms tumor who underwent surgical resection at the Children’s Hospital of Chongqing Medical University. Chemoresistance was defined as failure to achieve complete remission (CR) or partial remission (PR) with residual viable tumor ≤ 50% after 4 cycles of standard chemotherapy, or disease progression (PD) during chemotherapy, as evaluated by postoperative pathological examination and imaging (CT/MRI) according to the RECIST 1.1 criteria. Written informed consent was obtained from the legal guardians of all patients prior to specimen acquisition. This study was carried out in strict compliance with the ethical principles outlined in the Declaration of Helsinki and was approved by the Ethics Committee of the Children’s Hospital of Chongqing Medical University (Approval No.: 2025 − 174).

### Cell culture

WiT49 and THP-1 cell lines were obtained from the Cell Bank of the Chinese Academy of Sciences (Shanghai, China). The human bone marrow mesenchymal stem cell (BMSCs) line was purchased from the Beijing Union Cell Bank (Beijing, China). WT-CLS1 cells were generously provided by the Capital Institute of Pediatrics (Beijing, China). WiT49, WT-CLS1, and BMSCs were maintained in high-glucose Dulbecco’s Modified Eagle Medium (DMEM) containing 10% fetal bovine serum (FBS; Gibco, USA), 100 U/mL penicillin, and 100 µg/mL streptomycin. For BMSCs, exosome-depleted high-glucose DMEM was used, where FBS was pre-treated via ultracentrifugation to remove exosomes. THP-1 cells were cultured in RPMI 1640 medium supplemented with 10% FBS (Gibco, USA). All cells were incubated at 37 °C in a humidified environment with 5% CO₂.

### Bioinformatics analysis

Sequencing data and corresponding clinical metadata of the Wilms tumor cohort (TARGET-WT) were downloaded from the TARGET database. Differential expression analysis between tumor tissues and adjacent non-tumor tissues was conducted using the EdgeR package to identify differentially expressed mRNAs. For prognostic evaluation, univariate Cox proportional hazards regression was performed using the survival package in R, with overall survival (OS) as the primary endpoint and statistical significance set at *P* < 0.05. The relative abundance of tumor-infiltrating immune cells (TIICs) in TARGET-WT samples was estimated via the CIBERSORT algorithm using normalized gene expression profiles. Weighted gene co-expression network analysis (WGCNA) was applied to construct gene co-expression modules and identify modules linked to M2 macrophage infiltration. All bioinformatics results were visualized using R software with packages such as ggplot2 and pheatmap.

### Reagents and antibodies

Reagents and antibodies utilized in this study included: Phorbol 12-myristate 13-acetate (PMA; HY-18739, MCE, USA); Recombinant Human IL-4 (#200-04, PeproTech, USA); Recombinant Human IL-13 (#200 − 13, PeproTech, USA); D-Luciferin potassium salt (HY-12591B, MCE, USA); Tumor necrosis factor-α (TNF-α; HY-P1875, MCE, USA); IKK 16 (HY-13687, MCE, USA); CXCL17 (HY-P71878, MCE, USA); Rhodamine-WGA (MP6326-1MG, MKbio, China); Anti-CD163 (6646-1-AP, Proteintech, China); Anti-SNRPC (ab192028, Abcam, UK); Anti-PSMA4 (R25489, Zen BioScience, China); Anti-PPIH (11651-1-AP, Proteintech, China); Anti-PFDN4 (16045-1-AP, Proteintech, China); Anti-CKS1B (10610-1-AP, Proteintech, China); Anti-GAPDH (81640-5-RR, Proteintech, China); Anti-phospho-IKKα/β (#2697, CST, USA); Anti-IκBα (R23322, Zen BioScience, China); Anti-phospho-IκBα (#2859, CST, USA); Anti-NF-κB p65 (310099, Zen BioScience, China); Anti-phospho-NF-κB p65 (#3033, CST, USA); Anti-CXCL17 (18108-1-AP, Proteintech, China); Anti-CD9 (20597-1-AP, Proteintech, China); Anti-TSG101 (28283-1-AP, Proteintech, China); Anti-CD63 (25682-1-AP, Proteintech, China); Anti-calnexin (10427-2-AP, Proteintech, China).

### Cell transfection

SNRPC-shRNA lentivirus (Target sequence: shSNRPC#1, 5′-GCATTTCAACAAGGAAAGATA-3′; shSNRPC#2, 5′-CCAGACAGATAAGGATAGAGG-3′; shSNRPC#3, 5′-CCATCTGTGAGAAAGACACAC-3′;) and SNRPC overexpression lentivirus were acquired from Generulor Medical Technology Co., Ltd. (Zhuhai, China). Transfection and selection procedures were executed strictly following the manufacturer’s guidelines. Small interfering RNA (siRNA) targeting SNRPC (siRNA-SNRPC, siRNA-PSMA4, siRNA-PPIH, siRNA-PFDN4, siRNA-CKS1B) was synthesized by TSINGKE Biological Technology (Beijing, China) and delivered into cells using Lipo6000 reagent (Cat. No. C0526, Beyotime, Shanghai, China) according to the manufacturer’s protocol. Sequences of siRNAs are provided in Supplementary Table 1.

### Quantitative real-time polymerase chain reaction (qRT-PCR)

TRIzol reagent (Invitrogen, USA) was used for total RNA extraction from clinical specimens or cultured cells. First-strand cDNA was synthesized from 1 µg of total RNA using the PrimeScript™ RT Reagent Kit (Takara, Japan) following the manufacturer’s instructions. qRT-PCR was conducted with TB Green^®^ Premix Ex Taq™ II (Takara, Japan) on a 7500 Real-Time PCR System (Applied Biosystems, USA). Thermal cycling conditions were as follows: initial denaturation at 95 °C for 30 s; 40 cycles of denaturation at 95 °C for 5 s and annealing/extension at 60 °C for 34 s. GAPDH was used as the internal reference gene, and relative gene expression levels were calculated using the 2⁻ΔΔCt method. Primer sequences are listed in Supplementary Table 2.

### Western blot analysis

Cells were collected and lysed in RIPA buffer (Beyotime, Shanghai, China) supplemented with protease inhibitors (MCE, USA) on ice for 30 min. Lysates were centrifuged at 20,000×g for 30 min at 4 °C to obtain supernatants. For the detection of secretory protein CXCL17, the cell culture supernatant of each group was collected, and the proteins in the supernatant were concentrated by ultrafiltration tubes (molecular weight cut-off: 3 kDa) according to the manufacturer’s instructions. The same volume of concentrated supernatant protein from each group was subjected to SDS-PAGE electrophoresis, and Coomassie blue staining was used for protein loading alignment. Protein concentration was measured using a BCA Protein Assay Kit (Beyotime, Shanghai, China). Equal amounts of protein (10 µg) were separated by 8% SDS-PAGE and transferred onto polyvinylidene fluoride (PVDF) membranes (Millipore, USA). Membranes were blocked with NcmBlot Blocking Buffer (Cat. No. P30500, NCM Biotech, Suzhou, China) for 10 min at room temperature, then incubated with primary antibodies (1:1000 dilution) overnight at 4 °C. After washing with TBST, membranes were incubated with HRP-conjugated secondary antibodies (1:5000 dilution) for 2 h at room temperature. Protein bands were visualized using an ECL chemiluminescence kit (Affinity, China) and imaged with a Gel Doc XR+ system (Bio-Rad, USA). Band intensities were quantified using ImageJ software.

### Immunofluorescence (IF) staining

#### Tissue section staining

Tissue sections were baked at 60 °C for 2 h, dewaxed in xylene, and hydrated through a graded ethanol series. Antigen retrieval was carried out in citrate buffer (pH 6.0) at 98 °C for 15 min, followed by blocking with 3% BSA for 1 h at room temperature. Sections were incubated with primary antibodies (1:200 dilution) overnight at 4 °C. After washing with PBST, sections were stained using the Triple-Label Four-Color/Double-Label Three-Color Multiplex Fluorescence Staining Kit (AiFang Biological), including steps of Polymer-HRP secondary antibody incubation, TSA fluorochrome staining, and antibody elution, repeated per the manufacturer’s instructions until all target antibodies were probed. Nuclei were counterstained with DAPI, and images were captured using a fluorescence microscope (Canon, Japan) and analyzed with ImageJ.

#### NF-κB nuclear translocation assay

Cell coverslips were processed using the NF-κB Activation-Nuclear Translocation Detection Kit (SN371, Beyotime, Shanghai, China) according to the manufacturer’s protocol. Nuclear localization of NF-κB p65 was visualized using a fluorescence microscope (Canon, Japan), and the nuclear-to-cytoplasmic fluorescence intensity ratio was quantified using ImageJ.

#### Measurement of migrasomes

To assess migrasome formation, 35 mm confocal dishes were pre-coated with fibronectin (10 mg/mL) at 37 °C for 1 h. Cells were seeded in these fibronectin-coated dishes and cultured for 12–48 h. After incubation, cells were fixed with glutaraldehyde and stained with rhodamine-conjugated wheat germ agglutinin (Rhodamine-WGA; MP6326-1MG, MKbio). Migrasomes were visualized and analyzed using a confocal laser scanning microscope (CSU-W1 SoRa, Nikon, Japan).

#### Cell counting Kit-8 (CCK-8) assay

Tumor cells were seeded in 96-well plates at a density of 5 × 10³ cells per well, with 3 replicate wells per group. After treatment, 10 µL of CCK-8 solution (HY-K0301, MCE, USA) was added to each well, and plates were incubated at 37 °C for 2 h. Absorbance at 450 nm was measured using a microplate reader (Thermo Fisher Technologies, USA) to calculate cell viability.

### Flow cytometry analysis

THP-1 cells were differentiated into macrophages by treatment with 150 ng/mL PMA (MCE, USA) for 24 h, then co-cultured with WT cells in fresh medium or via a 0.4-µm Transwell system for 72 h. Macrophages were collected, washed with PBS, and stained with BV421-conjugated anti-human CD68 (564943, BD Pharmingen, USA) and APC-conjugated anti-human CD206 (561763, BD Pharmingen, USA) for 30 min at 4 °C in the dark. Cells were analyzed using a CytoFLEX LX flow cytometer (Beckman, USA), and the proportion of CD68⁺CD206⁺ cells was quantified using FlowJo software (version 10.4, USA).

### Migration and invasion assays

#### Migration assay

Transwell chambers (8 μm pore size, Corning, USA) were employed. 100 µL of serum-free medium containing 2 × 10⁴ cells was added to the upper chamber, and 500 µL of complete medium (10% FBS) was added to the lower chamber. After 48 h of incubation at 37 °C, non-migrated cells in the upper chamber were removed with a cotton swab. Migrated cells were fixed with methanol, stained with crystal violet, and counted in 5 random fields using an inverted microscope (Canon, Japan).

#### Invasion assay

The upper chamber membrane was pre-coated with 100 µL Matrigel (Corning, USA) and polymerized at 37 °C for 30 min. Subsequent steps were identical to the migration assay, and invasive cells were counted similarly.

#### Colony formation assay

Cells were seeded in 12-well plates at a density of 400 cells per well and cultured for 2 weeks. Colonies were stained with 0.5% crystal violet, photographed, and counted manually.

#### Dual-luciferase reporter gene assay

Potential binding sites of phosphorylated NF-κB p65 (p-P65) on the CXCL17 promoter were predicted using JASPAR (http://jaspar.genereg.net/). The CXCL17 promoter fragment was inserted into the pGL3 vector, and p-P65 was cloned into the pcDNA3.1–3×Flag vector. The pGL3-CXCL17 or pGL3-basic luciferase plasmid was co-transfected into HEK-293T cells with the p-P65 eukaryotic expression vector (containing r-p-P65 or pcDNA3.1–3×Flag) and the pGL-TK Renilla luciferase vector (internal reference). Luciferase activity was evaluated using the Dual-Luciferase Reporter Gene Assay System (Yeasen, China).

#### Chromatin immunoprecipitation (ChIP)-qPCR

ChIP assays were performed using the ChIP Kit (Cat. No. BW2501, Baiwei Biotechnology, Guangzhou, China). Briefly, 2–5 × 10⁷ WiT49 and WT-CLS1 cells were collected, cross-linked with 1% formaldehyde at room temperature for 10 min, and the reaction was terminated with 1.375 M glycine. Nuclei were isolated via gradient lysis, and genomic DNA was sheared into 200–600 bp fragments using a Diagenode UCD-300 sonicator (verified by agarose gel electrophoresis). Protein A/G magnetic beads were incubated overnight with ChIP antibodies or rabbit IgG (Proteintech, Cat. No. 30000-0-AP) as a negative control. Sonicated chromatin supernatants (1.0 mL for IP group, 0.4 mL for IgG group) were added to the bead-antibody complexes and incubated at 4 °C for 2–4 h; 0.1 mL of supernatant was reserved as the Input group. After washing, elution, and overnight de-crosslinking at 65 °C, DNA was purified by phenol-chloroform-isoamyl alcohol extraction and ethanol precipitation following RNase A and proteinase K treatment. qPCR was performed using Vazyme ChamQ SYBR qPCR Master Mix (Cat. No. Q311) on a Bio-Rad CFX96 instrument. The relative enrichment of p-p65 at CXCL17 promoter sites 1–3 was calculated using the 2⁻ΔΔCt method. Primer sequences are provided in Supplementary Table 3.

#### NOD/SCID mouse xenograft model

Animal study was approved by the Laboratory Animal Welfare and Ethics Committee of the Children’s Hospital of Chongqing Medical University (Approval No.: CHCMU-IACUC20250429002). Four/five-week-old male NOD/SCID mice were purchased from Hunan SLKJD Laboratory Animal Co., Ltd. (Hunan, China). Mice were housed in a specific pathogen-free (SPF) facility with controlled temperature (21 ± 2 °C), humidity (50 ± 10%), and a 12 h light/dark cycle, with free access to food and water.

#### Orthotopic renal tumor model

Mice were anesthetized with pentobarbital sodium, and the left dorsal renal area was depilated and disinfected. A small incision was made to expose the kidney, and 1 × 10⁶ WiT49 cells were slowly injected into the renal upper pole. The incision was sutured, and mice were monitored postoperatively. At the end of the experiment, the mice were euthanized, and the kidney tissues with orthotopic tumors were collected and weighed in full. The tumor tissue was closely integrated with the renal parenchyma and could not be separated and weighed alone, so the ratio of tumor tissue weight to renal tissue weight (tumor/renal tissue weight ratio) was calculated to reflect the relative tumor burden of the mice.

#### Lung metastasis model

5 × 10⁵ luciferase-expressing WiT49 cells were resuspended in 100 µL PBS and injected via the tail vein. Starting on day 7 post-injection, D-luciferin potassium salt (150 mg/kg; MCE, USA) was administered intraperitoneally every 2 days, and lung metastasis nodules were monitored using an AniView Pro in vivo imaging system. When the average surface photon flux reached ≈ 20,000 photons·s⁻¹·cm⁻², mice were randomized into groups (*n* ≥  5 per group) and treated via intravenous injection. Tumor growth was monitored continuously to evaluate therapeutic efficacy.

### Construction and characterization of BMSCs exosomes, LNP-siRNA, hEVs, and hEVs-DOX

#### Isolation of BMSCs exosomes

BMSCs were cultured in exosome-free DMEM for 48 h, and supernatants were collected. Supernatants were centrifuged sequentially at 300×g for 10 min, 2000×g for 20 min, and 10,000×g for 30 min at 4 °C to remove debris and dead cells. Exosomes were isolated by ultracentrifugation at 100,000×g for 70 min at 4 °C, resuspended in PBS, and stored at -80 °C.

#### Preparation of LNP-siRNA

LNP-siRNA was prepared using a microfluidic mixing approach: the aqueous phase (siRNA duplexes) was rapidly mixed with the ethanol phase (lipid mixture containing DLin-MC3-DMA, DSPC, cholesterol, and DMG-PEG2000 at a molar ratio of 50:10:38.5:1.5) at a 3:1 volume ratio. Buffer was exchanged to Tris/sodium citrate buffer (pH 7.4) via ultrafiltration/diafiltration, and the product was sterilized with a 0.2 μm filter. Particle size (z-avg) and zeta potential were measured using a Zetasizer (Malvern Panalytical, UK). siRNA encapsulation efficiency was determined using the Ribogreen assay.

#### Preparation of hEVs

hEVs were prepared using a pH-dependent fusion method as reported by Alena Ivanova et al. [23]: exosomes and LNPs were mixed at a 2:1 particle ratio in MES buffer (10 mM MES, 145 mM NaCl, 5 mM KCl, pH 5.5) and incubated at room temperature for 30 min. pH was adjusted to 7.4 with PBS, and hEVs were collected by ultracentrifugation at 100,000×g for 60 min at 4 °C. Empty hEVs, siNC@hEVs (loaded with negative control siRNA), and siSNRPC@hEVs (loaded with siSNRPC) were prepared separately.

#### Preparation of hEVs-DOX

Doxorubicin (DOX) was encapsulated into hEVs using the ammonium sulfate gradient method to generate DOX/siNC@hEVs and DOX/siSNRPC@hEVs, which were stored at -80 °C [24, 25].

### Characterization

Particle morphology was observed using transmission electron microscopy (Hitachi HT7700, Japan). Concentration and size distribution were measured with a NanoSight NS300 (Malvern, UK). Surface charge was determined using a Zetasizer ZSP (Malvern, UK). Expression of exosomal markers (ALIX, CD63, TSG101) and the negative marker calnexin was verified by Western blot.

### Drug loading and release assays

A standard curve for DOX was established using a multimode microplate reader (Thermo Fisher Technologies, USA) at excitation/emission wavelengths of 480/594 nm, leveraging DOX’s intrinsic fluorescence. Loading efficiency (LE) and encapsulation efficiency (EE) were calculated through DOX concentration gradient experiments. For release studies, hEVs-DOX was incubated in pH 7.4 or pH 5.5 buffer at 37 °C, and DOX concentration was measured at various time points to calculate cumulative release rates.

### Statistical analysis

All experiments were independently repeated at least 3 times, with data expressed as mean ± standard deviation (x ± s). GraphPad Prism v9 software (GraphPad Software, USA) was used for statistical analyses. Comparisons between two groups were analyzed using Student’s t-test; multiple group comparisons were performed using one-way ANOVA followed by Tukey’s post hoc test. Pearson correlation analysis was applied to assess the association between CD163 and SNRPC expression in Wilms tumor patients. Statistical significance was defined as *p < 0.05, **p < 0.01, and ***p < 0.001,.ns (not significant) *p* > 0.05.

## Results

### Higher proportion of M2-type TAMs in the tumor microenvironment of Wilms tumor patients with chemoresistance

**Fig. 1:**
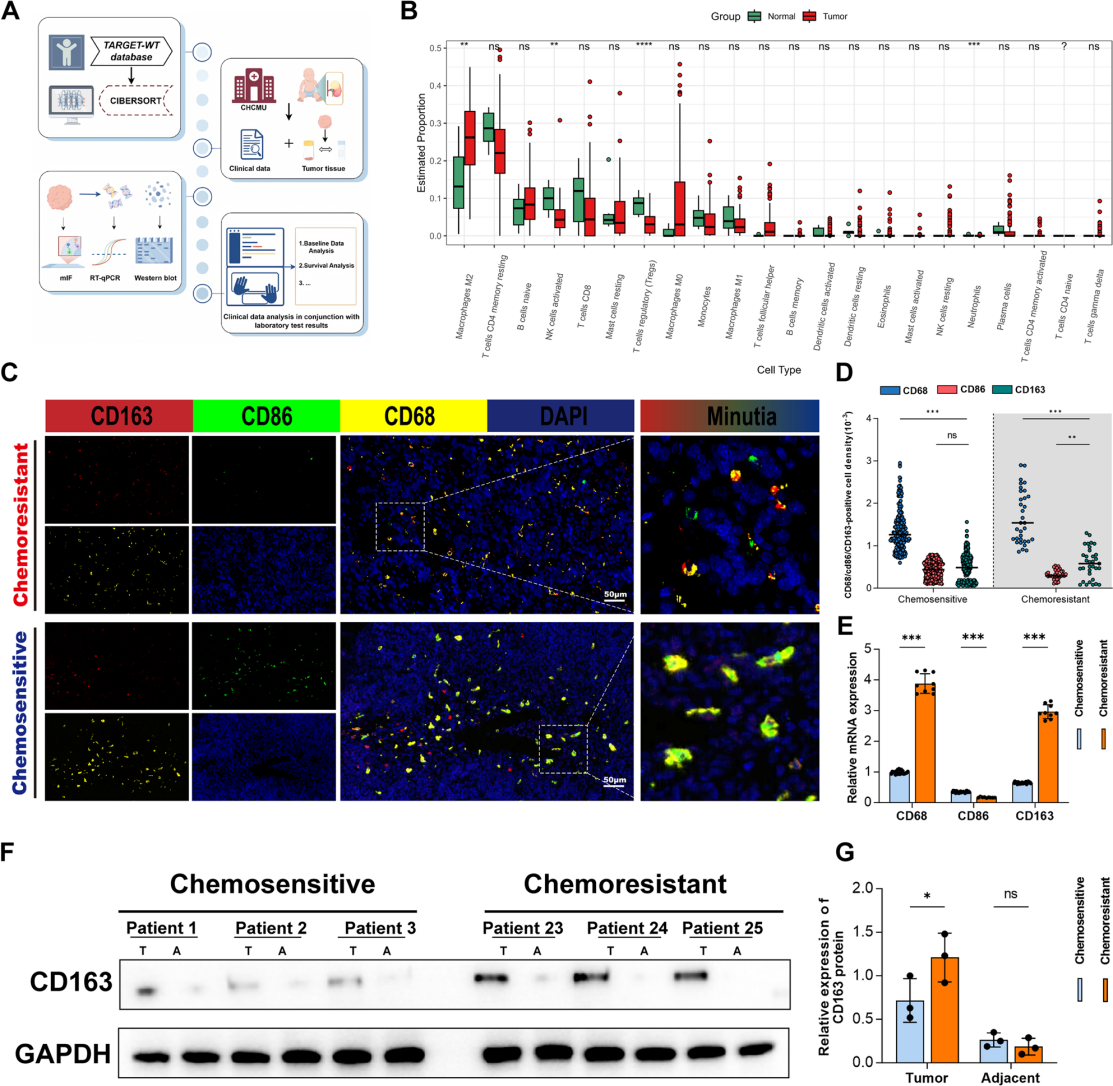
High infiltration of M2-type TAMs correlates with chemoresistance and poor prognosis in Wilms tumor. **A** Flow Chart of the Research Procedure. **B** Distribution of immune cell infiltration in tumor and adjacent non-tumor tissues from the TARGET-WT dataset. **C**, **D** Multiplex immunofluorescence staining showing the expression of CD68, CD86, and CD163 in 321 clinical Wilms tumor (WT) specimens (scale bar = 50 μm). **E**-**G** qRT-PCR and Western blot (WB) analyses of CD163 expression in 25 fresh WT tissues collected from our hospital. *P < 0.05, **P < 0.01, ***P < 0.001

The relative abundance of tumor-infiltrating immune cells (TIICs) in TARGET-WT samples was analyzed, and a comprehensive evaluation was conducted using clinical data and pathological tissues from Wilms tumor (WT) patients treated in our center (Fig. 1A). TARGET-WT data analysis revealed that M2-type TAMs were the most abundant type of immune cells infiltrating the WT tumor microenvironment. Furthermore, the infiltration of M2-type TAMs in tumor tissues was significantly higher than that in adjacent non-tumor tissues (Fig. 1B). Subsequently, clinical information of 321 WT patients diagnosed by surgery in our hospital was summarized (Table 1), and multiplex immunofluorescence staining was performed on tumor tissue sections of these patients. CD68 + cells were identified as total macrophages, CD68 + CD163+ cells as M2 macrophages, and CD68 + CD86+ cells as M1 macrophages. Three digital images were randomly selected from each entire tissue section. The number of fluorescence signal-positive cells was counted, and the proportion relative to the total number of cells was determined (Fig. 1C). Results showed that the proportion of M2-type TAMs was significantly higher than that of M1-type TAMs in the chemoresistant group (Fig. 1D). Finally, RT-qPCR and Western blot (WB) detection were performed on 25 fresh WT tumor tissues obtained clinically, and the results also showed a higher proportion of M2-type TAMs infiltration in tumor tissues of patients in the chemoresistant group (Figs. 1E-G). Therefore, we found that tumor tissues of patients with chemoresistance had a higher proportion of M2-type TAMs infiltration, and M2-type TAMs were associated with chemoresistance in WT patients.

**Table 1:** Demographic and clinicopathological variables of WT patients

Variable	Group		*p*-value^2^
	Chemosensitive *N* = 288^1^	Chemoresistant *N* = 33^1^	
Age			0.243
Median (Q1, Q3)	24.5 (13.0, 47.0)	36.0 (12.0, 56.0)	
Gender, *n*(%)			0.723
1	139 (48)	17 (52)	
2	149 (52)	16 (48)	
Side, *n*(%)			0.886
1	142 (49)	17 (52)	
2	137 (48)	16 (48)	
3	9 (3.1)	0 (0)	
Stage, *n*(%)			0.008
1	47 (16)	3 (9.1)	
2	80 (28)	6 (18)	
3	116 (40)	13 (39)	
4	29 (10)	11 (33)	
5	16 (5.6)	0 (0)	
Tumorsize			0.156
Median (Q1, Q3)	11.0 (9.0, 14.0)	10.0 (7.0, 14.0)	
Metastasis, *n*(%)			< 0.001
0	279(97)	21(64)	
1	9 (3.1)	12 (36)	
Neoadjuvantchemotherapy, *n*(%)			0.447
0	210(73)	22(67)	
1	78 (27)	11 (33)	
Pathologicaltype, *n*(%)			0.785
1	230 (80)	26 (79)	
2	33 (11)	5 (15)	
3	25 (8.7)	2 (6.1)	
Survivaltime, Months			< 0.001
Median (Q1, Q3)	182.0 (168.0, 194.0)	28.0 (16.0, 175.0)	
Death, *n*(%)			< 0.001
0	277(96)	14(42)	
1	11 (3.8)	19 (58)	
Mtotal			0.002
Median (Q1, Q3)	1.263 (0.989, 1.562)	1.538 (1.148, 2.157)	
M2			< 0.001
Median (Q1, Q3)	0.460 (0.149, 0.656)	0.947 (0.821, 1.056)	
M1			0.247
Median (Q1, Q3)	0.432 (0.262, 0.611)	0.382 (0.279, 0.466)	
M2/M1			< 0.001
Median (Q1, Q3)	1.243 (0.244, 2.312)	2.741 (1.971, 3.316)	

1 Median (IQR) or Frequency (%)

2 Wilcoxon rank sum test; Pearson’s Chi-squared test; Fisher’s exact test

### M2-type TAMs promote doxorubicin chemoresistance in vitro and in vivo models

**Fig. 2:**
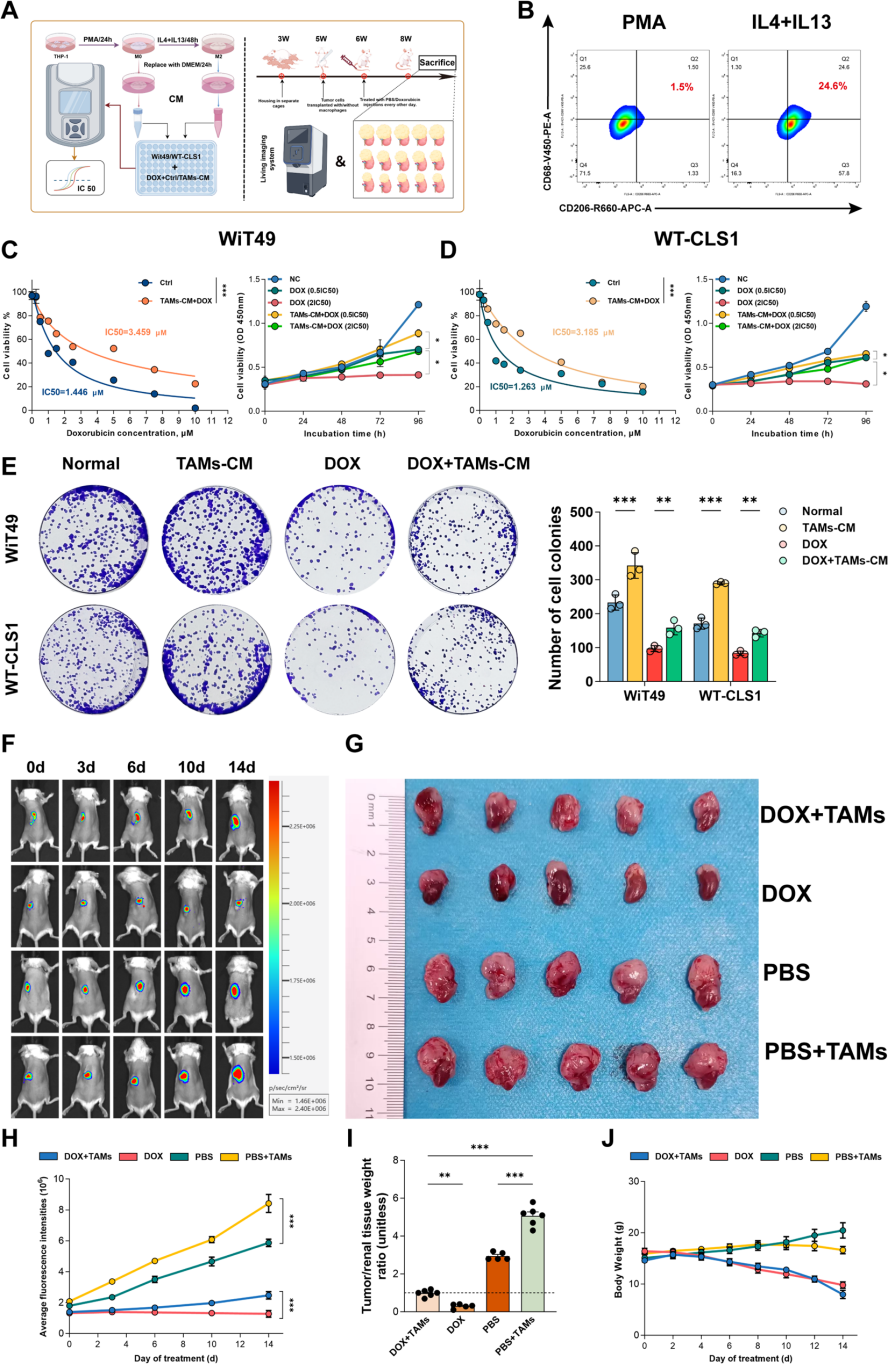
M2-type TAMs promote chemoresistance in Wilms tumor cell lines in vitro and in vivo. **A** Schematic diagram of in vitro and in vivo experimental design. **B** Flow cytometry analysis confirming the polarization of THP-1 cells into M2-type macrophages induced by IL-4 and IL-13. **C**, **D** IC₅₀ values of WiT49 and WT-CLS1 treated with DOX in the presence or absence of M2-TAMs conditioned medium, as well as the effects of different concentrations of DOX on cell viability at multiple time points. **E** Colony Formation Assay: Evaluation of Clonogenic Capacity of WiT49 and WT-CLS1 Cells in Response to DOX Treatment With or Without M2-TAMs Conditioned Medium Intervention. **F**, **H** In vivo imaging monitoring the growth of orthotopic tumors and quantification of fluorescence intensity during animal experiments. **G**, **I** Representative images and relative weight statistics of orthotopic tumors after doxorubicin treatment in mice with or without TAMs co-transplantation(First-time use of the renal orthotopic tumor model; 2 extra mice were included to avoid modeling failure. All models succeeded, and the 2 mice were randomly assigned to DOX+TAMs/PBS+TAMs groups (1 each), resulting in 6 mice for these two groups vs. 5 for DOX/PBS groups. Consistent experimental conditions and statistical analysis with actual sample sizes ensure result reliability; Tumor tissue was closely integrated with renal parenchyma and unable to be dissected and weighed independently; the tumor/renal tissue weight ratio was used to reflect the relative tumor burden of mice, and this indicator has no physical unit). **J** Body weight changes of mice during the experiment

To further verify whether M2-type TAMs can promote chemoresistance in WT, we designed in vitro and in vivo experiments for validation (Fig. 2A). In vitro, co-culture experiments were conducted using conditioned media from different types of macrophages and tumor cells. First, THP-1 cells were induced to adherent cells with PMA, then the medium was removed and the cells were divided into two groups: one group was supplemented with normal medium, and the other with M2 macrophage induction system containing IL-4 and IL-13. After continuous culture for 48 h, the culture supernatants of the two groups were collected separately and co-cultured with WiT49 and WT-CLS1 cells, followed by doxorubicin (DOX) intervention. Different DOX concentrations were set to determine the half-maximal inhibitory concentration (IC₅₀) of the two cell lines under DOX intervention, detect cell viability at multiple time points, and perform colony formation assay to evaluate the clonogenic capacity of tumor cells treated with M2-type TAM-conditioned medium under DOX intervention. Flow cytometry showed a significant increase in the proportion of M2-type macrophages after induction with IL-4 and IL-13 (Fig. 2B). There were significant differences in the IC₅₀ values of the two tumor cell lines treated with conditioned media, and M2-type TAM-conditioned medium significantly increased the IC₅₀ values of the two tumor cell lines. Meanwhile, M2-type TAM-conditioned medium significantly enhanced the drug resistance of the two tumor cell lines to high and low concentrations of DOX at multiple time points (Figs. 2C, D). In addition, colony formation assay indicated that M2-type TAM-conditioned medium significantly improved the clonogenic capacity of tumor cells under DOX intervention (Fig. 2E). For in vivo experiments, severely immunodeficient NOD/SCID mice were used to establish two orthotopic tumor models: tumor cells alone and tumor cells + macrophages. One week after tumor transplantation, in vivo imaging was performed to detect changes in tumor size, followed by intervention. Results showed that macrophages promoted tumor growth in the two non-chemotherapy groups, and partially attenuated the therapeutic effect of DOX in the DOX intervention group, promoting chemoresistance (Figs. 2F, H). The inhibitory effect of experimental interventions on renal orthotopic tumor growth was confirmed by both gross morphological observation (Fig. 2G) and quantitative analysis of tumor burden (Fig. 2I). The less pronounced visual difference in tumor size in the two-dimensional gross images (Fig. 2G) compared with the statistical results (Fig. 2I) is due to the stereoscopic structure of orthotopic tumors and shielding by renal parenchyma, which limit the ability of two-dimensional imaging to reflect actual tumor mass. In contrast, the tumor/renal tissue weight ratio in Fig. 2I is a quantitative index obtained by actual weighing, which objectively and accurately characterizes relative tumor burden, thus showing more prominent statistical differences. Notably, the two datasets exhibit consistent change trends, and their mutual verification further confirms the significant inhibitory effect of experimental interventions on renal orthotopic tumor growth. During the experiment, the body weight of mice in the DOX intervention group was lower than that in the non-administration group (Fig. 2J). Through in vitro and in vivo experiments, we further verified that M2-type TAMs promote tumor cell chemoresistance to doxorubicin.

### Tumor SNRPC gene is positively correlated with M2-type TAM marker gene CD163 expression, and associated with chemoresistance and prognosis

**Fig. 3:**
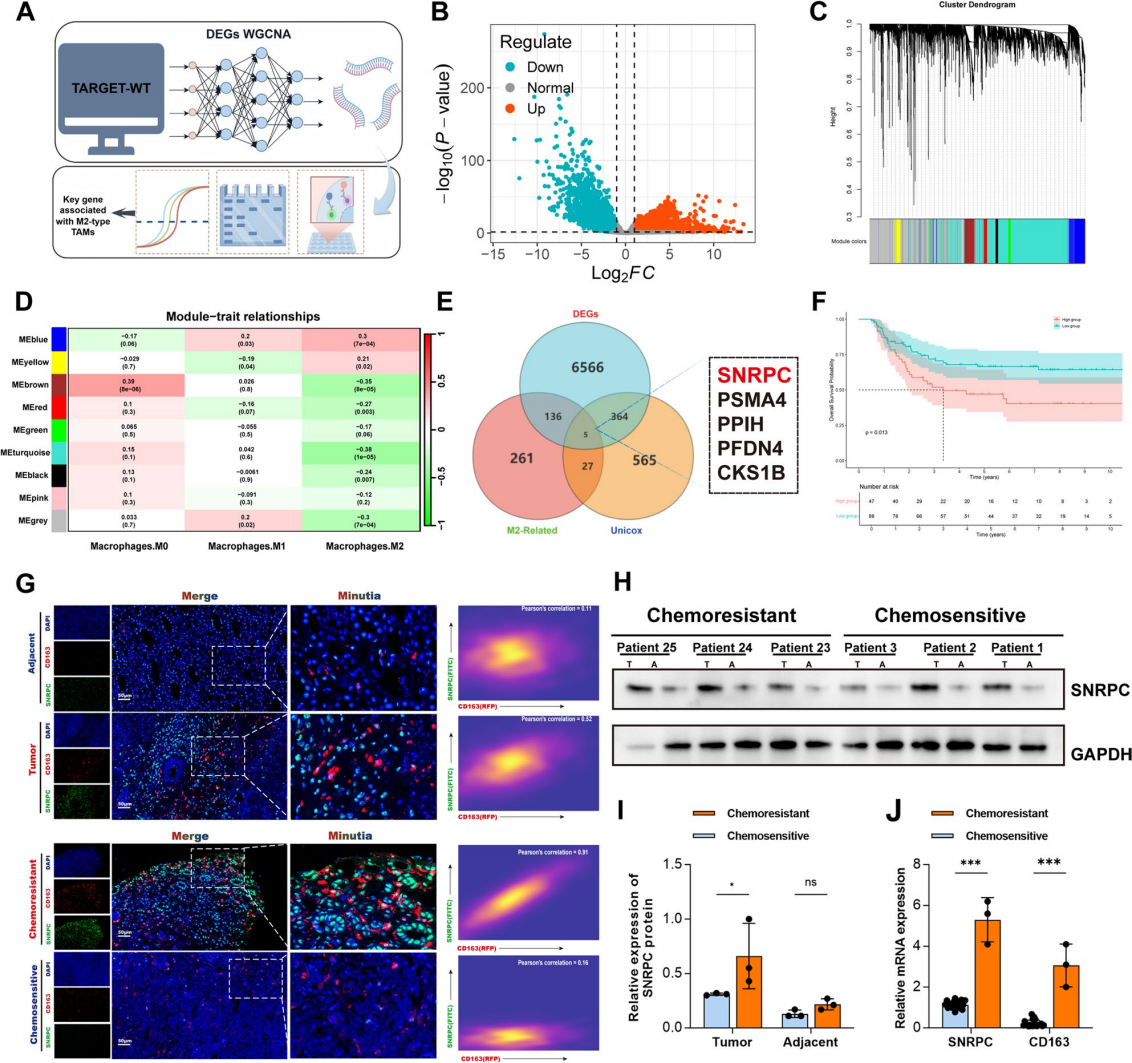
High SNRPC expression correlates with M2-type TAMs infiltration and predicts poor prognosis in clinical Wilms tumor specimens. **A** Flow Chart of the Research Procedure. **B** Volcano plot of differentially expressed genes (green indicates downregulated genes; red indicates upregulated genes). **C**, **D** WGCNA cluster dendrogram and module assignment using a dynamic tree-cutting algorithm. Correlation between module genes and immune cell infiltration: the abscissa represents different types of immune cell infiltration, the ordinate represents different modules, and each rectangle displays the Pearson correlation coefficient. **E** Venn diagram showing the overlap among upregulated genes, M2-type TAMs-associated genes, and poor-prognosis genes from the TARGET-WT sequencing dataset. **F** Kaplan-Meier survival curves based on SNRPC expression levels in the TARGET-WT dataset. **G** SNRPC expression in tumor vs. adjacent non-tumor tissues and its correlation with CD163 in clinical WT specimens (Pearson correlation analysis) and SNRPC expression in chemoresistant vs. non-chemoresistant WT specimens and its correlation with CD163 (Pearson correlation analysis). **H**-**J** WB and qRT-PCR analyses of SNRPC expression in 25 fresh WT tissues

Tumor cells recruit and drive M2 polarization of tumor-associated macrophages (TAMs) by releasing inflammatory cytokines and immunosuppressive metabolites, which dominate in various cancers. These cells not only promote tumor progression but also lead to chemoresistance. Therefore, our aforementioned study confirmed that M2-type TAMs promote chemoresistance. To further explore the regulatory mechanism between tumor cells and TAMs, we used weighted gene co-expression network analysis (WGCNA) to identify potential regulatory genes associated with M2-type TAMs in WT (Figs. 3A-D). By intersecting with differentially expressed genes and poor-prognosis genes, we identified five candidate potential regulatory genes (Fig. 3E). Survival analysis results showed that the SNRPC gene was a poor-prognosis gene (Fig. 3F). Subsequently, multiplex immunofluorescence staining of clinical tissue sections from our hospital and flow cytometry results of co-culturing macrophages with two tumor cell lines after knocking down each of the five candidate genes separately showed that: SNRPC gene expression in tumors was significantly higher than that in adjacent non-tumor tissues. In addition, compared with non-chemoresistant group sections, SNRPC gene was significantly highly expressed in the chemoresistant group and highly positively correlated with the expression of M2-type TAM marker gene CD163 (Fig. 3G), while the other four genes did not show such distribution differences (Fig. S1A). Flow cytometry results also confirmed that among the five candidate genes, only the SNRPC gene showed the potential to promote macrophage M2-TAM polarization (Fig. S1B). In addition, RT-qPCR and WB results of 25 clinical tissue sections also confirmed that SNRPC gene expression was significantly higher in the chemoresistant group than in the non-chemoresistant group (Figs. 3H-J).

### Tumor SNRPC gene affects macrophage M2-TAM polarization and proliferation, migration, and invasion activities of Wilms tumor cells

**Fig. 4:**
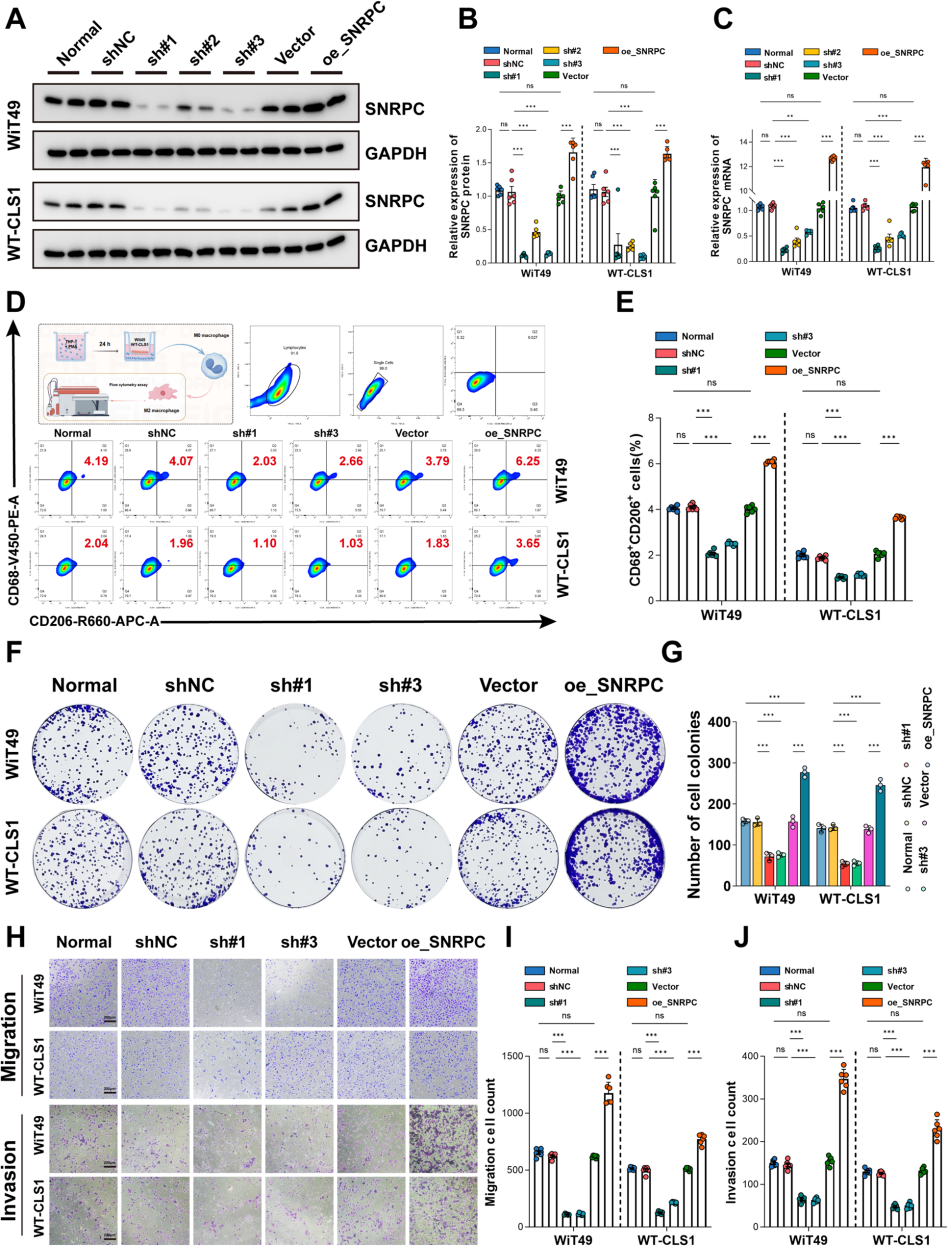
Tumor SNRPC Gene Affects Macrophage M2-TAM Polarization and Proliferation, Migration, and Invasion Activities of Wilms Tumor Cells. **A**-**C** WB and RT-qPCR analysis showing the expression of SNRPC in WT cells with SNRPC silencing (sh_SNRPC) or overexpression (oe_SNRPC). **D**, **E** Flow cytometry analysis of CD68⁺CD206⁺ macrophage proportion in the Transwell co-culture system, with WiT49/WT-CLS1 cells overexpressing (oe_SNRPC) or silencing SNRPC in the upper chamber and PMA-treated THP-1 cells in the lower chamber. **F**, **G** Colony Formation Assay: Effects of SNRPC Gene Knockdown or Overexpression on the Clonogenic Capacity of Two Tumor Cell Lines. **H**-**J** Transwell assays evaluating the migration and invasion abilities of WT cells with SNRPC silencing or overexpression

Stable knockdown and overexpression cell lines of the SNRPC gene, as well as their respective negative control cell lines, were successfully constructed in WiT49 and WT-CLS1 using lentiviral transfection and screening. WB and RT-qPCR results confirmed the successful construction of stably transfected cell lines (Figs. 4A-C). Flow cytometry results suggested that high expression of the SNRPC gene could promote macrophage M2-TAM polarization, while knockdown inhibited macrophage M2-TAM polarization (Figs. 4D, E). Colony formation assay (Figs. 4F, G) and Transwell assay (Figs. 4H-J) respectively confirmed that SNRPC gene knockdown significantly inhibited the proliferation, migration, and invasion activities of the two tumor cell lines, while overexpression of the SNRPC gene significantly promoted the proliferation, migration, and invasion activities of the two tumor cell lines.

### Tumor SNRPC gene drives M2-type TAM infiltration in WT to exert M2-type TAM-dependent chemoresistance

**Fig. 5:**
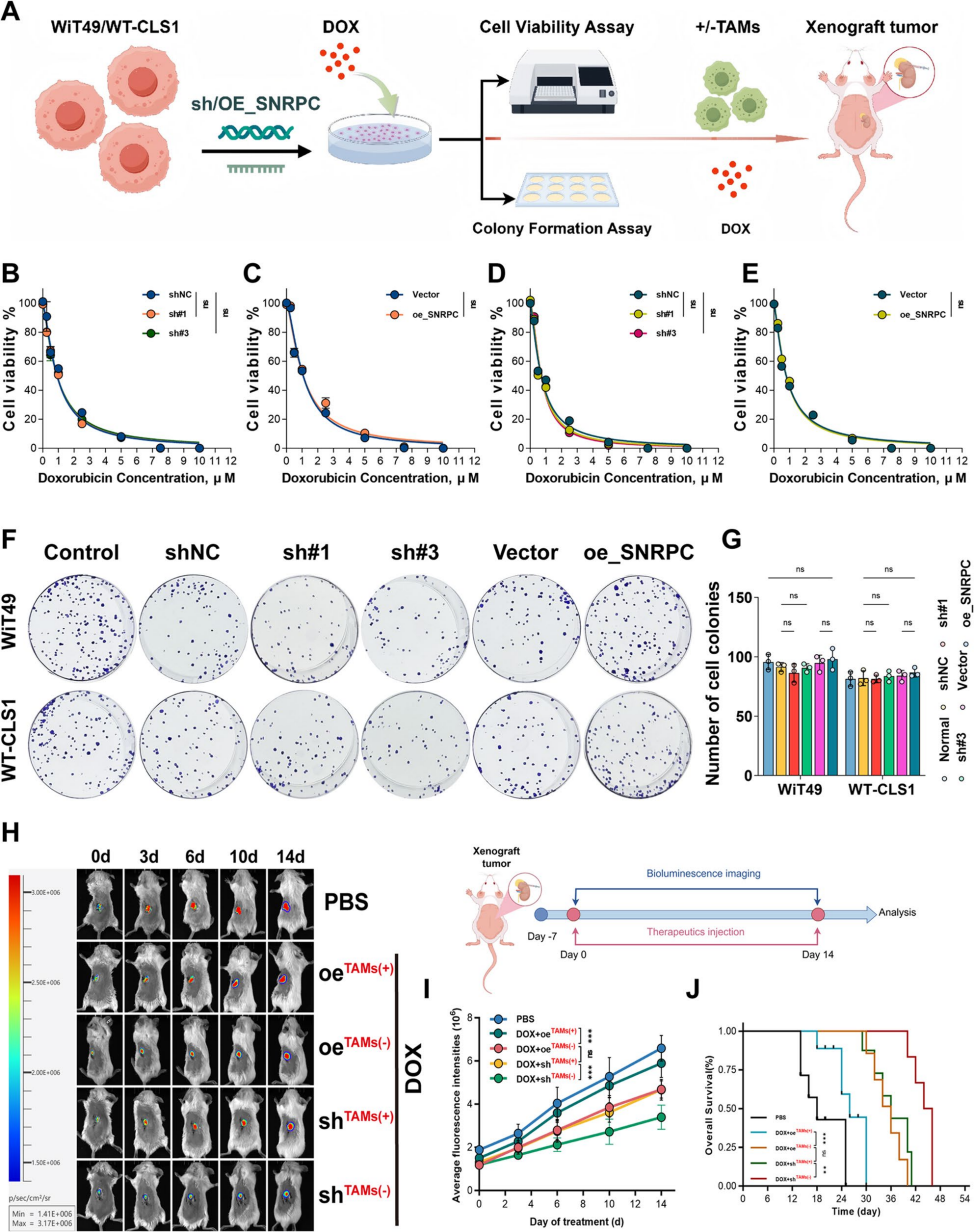
SNRPC promotes Wilms tumor cell proliferation and M2-type TAMs-dependent chemoresistance in vitro and vivo. **A** Flow Chart of the Research Procedure. **B**-**E** CCK-8 assays determining doxorubicin IC₅₀ values in WT cells with altered SNRPC expression. **F**, **G** Colony Formation Assay: Effects of SNRPC Gene Knockdown or Overexpression on the Clonogenic Capacity of Two Tumor Cell Lines Under DOX Treatment. **H** In vivo imaging monitoring the growth of orthotopic tumors formed by WiT49 cells (with or without SNRPC silencing) co-transplanted with or without TAMs after doxorubicin treatment. **I** Quantification of tumor fluorescence intensity during growth. **J** Kaplan-Meier survival curves of tumor-bearing mice

Subsequently, in vitro and in vivo experiments were conducted to further explore whether the tumor SNRPC gene can promote chemoresistance in WT cells (Fig. 5A). Interestingly, in the two tumor cell lines, neither knockdown nor overexpression of the SNRPC gene significantly changed the DOX IC₅₀ values of the two tumor cell lines (Figs. 5B-E). Colony formation assay also indicated that neither knockdown nor overexpression of the SNRPC gene significantly changed the clonogenic capacity of the two tumor cell lines under DOX intervention (Figs. 5F, G). This is inconsistent with the previous conclusion that the SNRPC gene promotes chemoresistance. Considering the complexity of the tumor microenvironment, especially whether the SNRPC gene interacts with the host immune system to mobilize other mechanisms to exert its anti-tumor therapeutic effect, it remains unclear. Given that the SNRPC gene is an M2-type TAM-related gene and previous experiments have confirmed that the SNRPC gene has the ability to regulate macrophage M2-type TAM polarization rate, we speculate that it may exert M2-type TAM-dependent chemoresistance by driving high infiltration of M2-type TAMs in WT. Therefore, we designed a conditional orthotopic tumor model, and experiments under conditional intervention showed M2-type TAM-dependent chemoresistance of the SNRPC gene (Figs. 5H, I). Kaplan-Meier survival curves revealed that TAMs significantly shortened the survival time of mice in both chemotherapy and non-chemotherapy groups (Fig. 5J).

### Tumor SNRPC positively regulates CXCL17 via NF-κB signaling to drive M2-type TAMs infiltration and chemoresistance in vitro

**Fig. 6:**
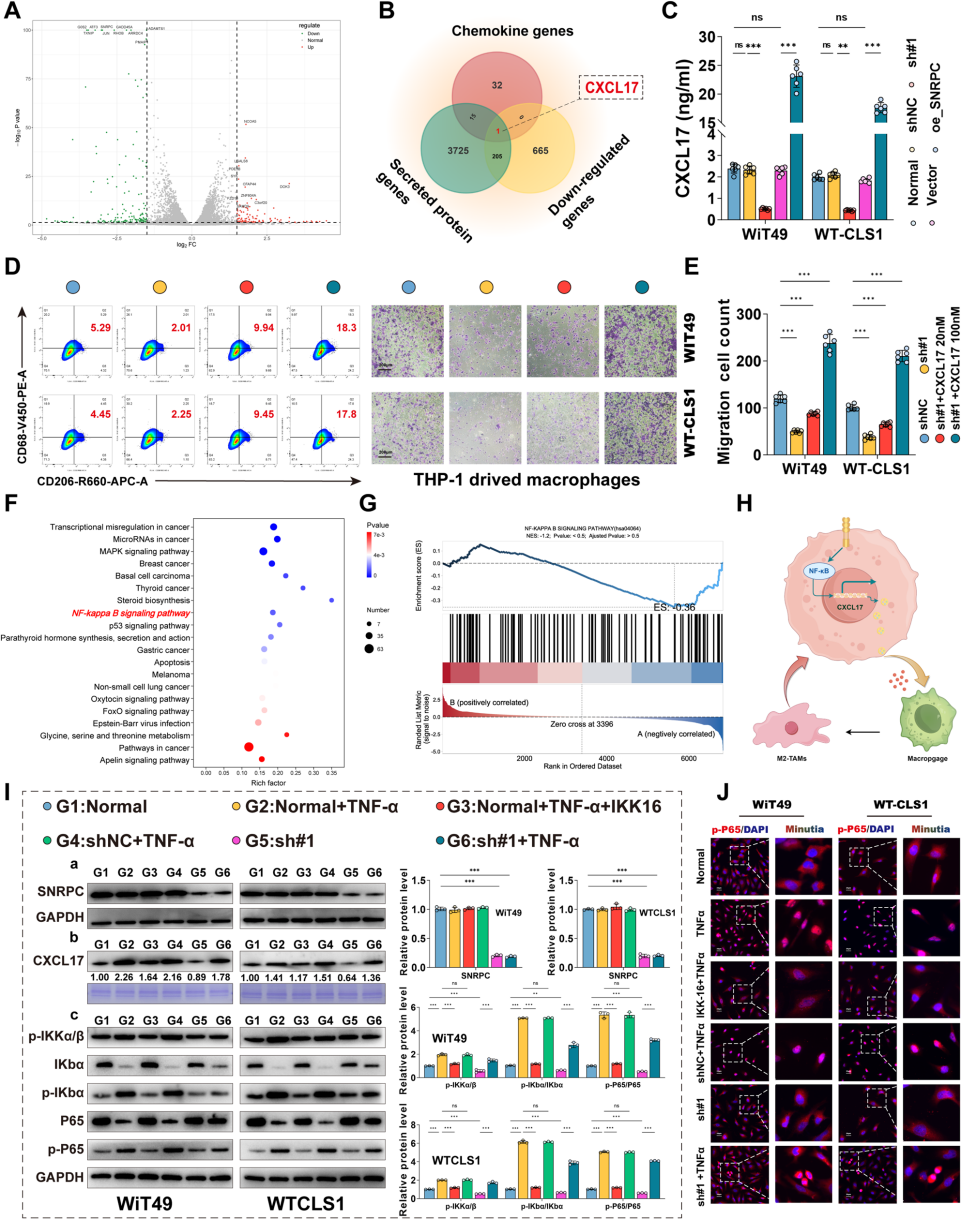
Tumor SNRPC Gene Positively Regulates CXCL17 via NF-κB Signaling Pathway to Drive M2-type TAM Infiltration in WT and Induce Chemoresistance in vitro. **A** RNA sequencing analysis of differentially expressed genes in WiT49 cells with stable SNRPC silencing vs. control cells. **B** Venn diagram showing the overlap among downregulated genes, secreted proteins, and chemokines. **C** ELISA analysis of CXCL17 concentration secreted by WT cells with SNRPC silencing (sh_SNRPC) or overexpression (oe_SNRPC). **D**, **E** Transwell migration assays of macrophages and Flow cytometry analysis of CD68⁺CD206⁺ macrophage proportionin the co-culture system, with SNRPC-silenced WiT49/WT-CLS1 cells and different doses of recombinant human CXCL17 in the lower chamber. **F** KEGG pathway enrichment analysis of differentially expressed genes. **G** GSEA analysis of the NF-κB signaling pathway. **H** Schematic Diagram: SNRPC Gene in Tumor Cells Regulates CXCL17 Secretion via the NF-κB Signaling Pathway to Affect TAMs Recruitment and Polarization. **I** SNRPC regulates CXCL17 via NF‑κB pathway. (6I‑a) SNRPC expression. GAPDH as control; overexpression verified in Figs. 4A, C. (6I‑b) Secreted CXCL17 in concentrated supernatants.(6I‑c) NF‑κB pathway proteins. GAPDH as control. **J** Immunofluorescence analyses of p-P65 nuclear translocation after treatment with TNF-ɑ (NF-κB agonist) or IKK-16 (IκB inhibitor) in SNRPC-silenced WiT49/WT-CLS1 cells

Chemokines are a class of small-molecule cytokines or signaling proteins secreted by cells, with a molecular weight of approximately 8–10 kDa, divided into four major subfamilies (CXC, CC, CX₃C, and XC) based on the arrangement of N-terminal cysteine residues. Their structural feature is a specific three-dimensional conformation formed by four conserved cysteine residues, which activates downstream signaling pathways by binding to G protein-coupled receptors to regulate the directional migration of immune cells. Although multiple studies have confirmed that various tumor cells recruit and drive M2 polarization of tumor-associated macrophages (TAMs) by releasing chemokines into the tumor microenvironment to further exert tumor-promoting effects and resist anti-tumor drug therapy, there are no such research reports in WT. To explore whether there is such a regulatory mechanism between the WT tumor SNRPC gene and M2-type TAMs, lentivirus-stably knockdown WiT49 cell line and untreated WiT49 cell line were subjected to transcriptome sequencing simultaneously (Fig. 6A). CXCL17 was identified after intersecting differentially expressed genes with secreted proteins and chemokine families (Fig. 6B). Previous studies have shown that CXCL17 is an important macrophage chemokine. In addition, CXCL17 can induce the recruitment of various myeloid cells and has chemotactic activity on inflammatory cells such as dendritic cells and monocytes. To verify whether the expression level of the SNRPC gene affects the expression level of CXCL17, ELISA detection of CXCL17 content in the culture system of SNRPC gene knockdown and overexpression in the two WT cell lines was performed (Fig. 6C). Results showed that knockdown of the SNRPC gene significantly reduced the concentration of CXCL17 in the culture system, while overexpression of the SNRPC gene significantly increased the concentration of CXCL17 in the culture system. The expression level of the SNRPC gene was positively correlated with the secretion level of CXCL17. Migration and flow cytometry results of co-culturing SNRPC gene knockdown cell lines of WiT49 and WT-CLS1 with THP-1 cells with different doses of recombinant human CXCL17 protein showed (Figs. 6D, E) that recombinant human CXCL17 protein partially rescued the reduced macrophage migration and M2-type TAM polarization rate caused by SNRPC gene knockdown in a dose-dependent manner. The intervention concentration of 100 nM promoted more macrophage migration and M2-type TAM polarization rate than 20 nM.

To study the regulatory pathway between the SNRPC gene and CXCL17, KEGG pathway enrichment analysis of transcriptome data showed significant differences in the NF-κB signaling pathway between the two groups (Fig. 6F). Further GSEA analysis suggested that the NF-κB signaling pathway was inhibited in the SNRPC gene knockdown group (Fig. 6G), indicating that high expression of the SNRPC gene may upregulate the NF-κB signaling pathway to regulate the tumor microenvironment (Fig. 6H). In addition, Western blot analysis confirmed (Figs. 6I-K) that the levels of p-IKKα/β, p-P65, and CXCL17 were decreased in SNRPC-knockdown WT cells, while significantly increased in the SNRPC overexpression group. These results indicate that highly expressed SNRPC can activate the NF-κB signaling pathway. The NF-κB agonist TNF-α partially reversed the inhibition of the NF-κB signaling pathway and the reduction of CXCL17 production caused by SNRPC knockdown. The NF-κB signaling pathway inhibitor (IKK-16) successfully blocked the activation of the NF-κB signaling pathway and high secretion of CXCL17 stimulated by high expression of SNRPC. The nuclear translocation fluorescence results of p-P65 in the two cell lines further indicated that the nuclear translocation level of p-P65 was decreased in SNRPC-knockdown WT cells, while the NF-κB agonist TNF-α partially reversed the decreased nuclear translocation level of p-P65 caused by SNRPC knockdown (Fig. 6L).

### Tumor SNRPC gene positively regulates CXCL17 via NF-κB signaling pathway to drive M2-type TAM infiltration in WT and induce chemoresistance in vivo

**Fig. 7:**
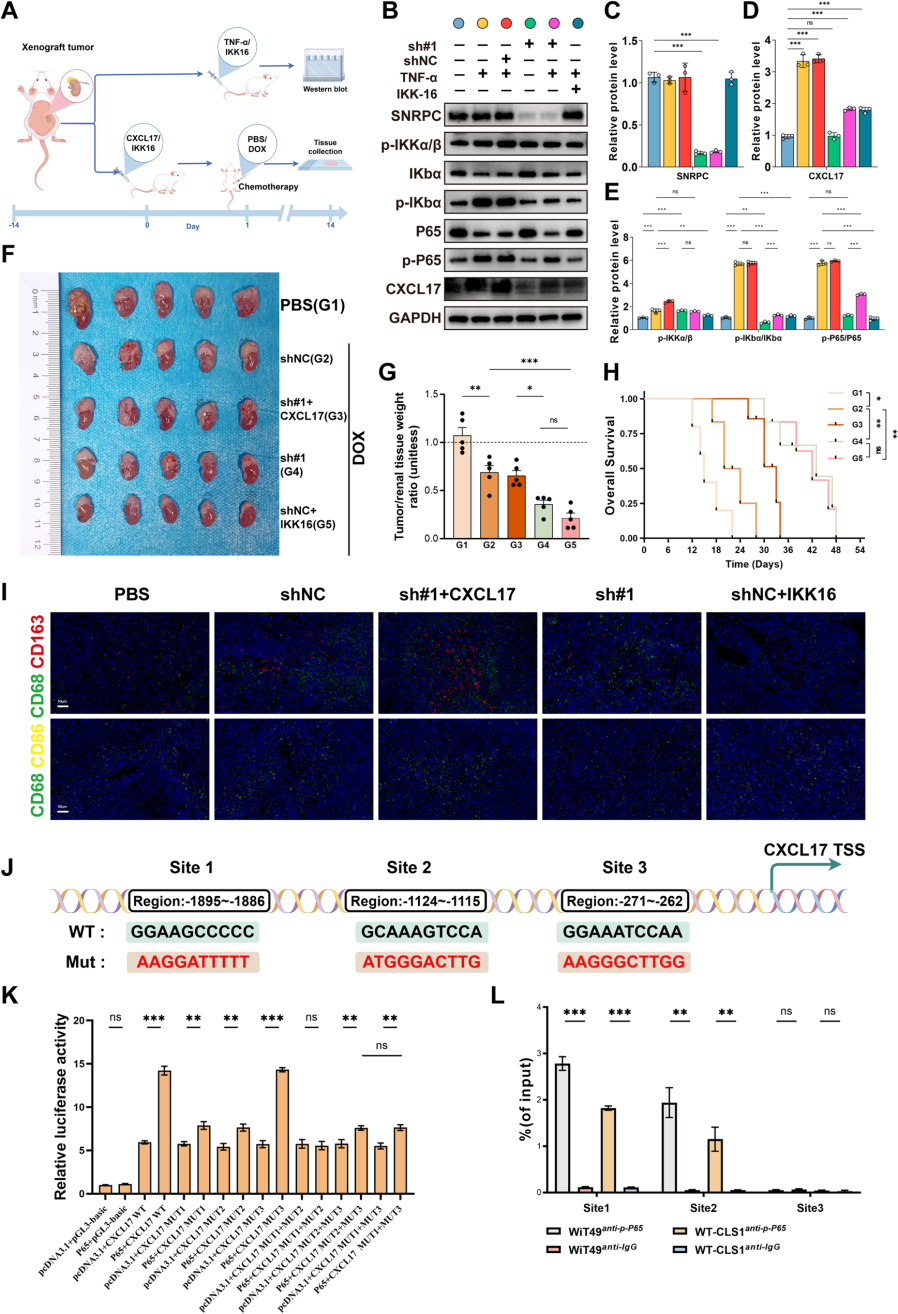
Tumor SNRPC Gene Positively Regulates CXCL17 via NF-κB Signaling Pathway to Drive M2-type TAM Infiltration in WT and Induce Chemoresistance in vivo. **A** Schematic diagram of the in vivo experiment. **B**-**E** WB analysis of protein expression changes in the SNRPC-NF-κB-CXCL17 regulatory axis in tumor tissues. **F**-**G** Representative images and relative volume statistics of orthotopic tumors formed by SNRPC-silenced WiT49 cells after different interventions. **H** Kaplan-Meier survival curves of mice in different treatment groups. **I** Immunofluorescence analysis of CD68⁺CD206⁺/CD68⁺CD86⁺ macrophage proportions in tumor tissues from different treatment groups. **J** Predicted binding sites of p-P65 to the CXCL17 promoter using the JASPER database. **K** Dual-luciferase reporter gene assay was applied to evaluate p-P65-mediated modulation of wild-type CXCL17 promoter transcriptional activity. **L** Chromatin immunoprecipitation quantitative polymerase chain reaction (ChIP-qPCR) assay was employed to determine the binding affinity of p-P65 to the CXCL17 promoter region

To further explore whether the regulatory pathway between the SNRPC gene and CXCL17 in vivo is consistent with that in vitro, we designed two in vivo experiments with different protocols. In the first group of orthotopic tumor models, WB detection was performed on tumor tissues after intervention under the same conditions as in vitro, while in the second group of in vivo experiments, detection of chemoresistance to DOX after conditional intervention was conducted (Fig. 7A). The two groups of in vivo experiments were combined to comprehensively evaluate the consistency of the regulatory mechanism found in vitro in vivo.

Compared with in vitro experimental results, in the first group of xenografts (Figs. 7B-E), the change trend of the SNRPC-NF-κB-CXCL17-M2-type TAMs signaling axis under conditional intervention was consistent with the in vitro experimental results, further confirming the impact of SNRPC on the activation of the NF-κB signaling pathway in vivo. The results of the second group of in vivo experiments suggested the chemoresistance to DOX exerted by the SNRPC-NF-κB-CXCL17-M2-type TAMs signaling axis (Figs. 7F-H). In addition, immunofluorescence detection of tumor tissue sections from the second group of experimental mice found that CXCL17 partially reversed the reduced M2-type TAM infiltration caused by SNRPC gene knockdown (Fig. 7I). Overall, SNRPC promotes the expression of CXCL17 in WT cells by activating the NF-κB pathway, driving M2-type TAM infiltration in WT and inducing chemoresistance.

However, whether p-P65 directly regulates CXCL17 transcription remains to be clarified. According to the JASPAR database, p-P65 can directly bind to the CXCL17 promoter (Fig. S2A). In addition, we used AlphaFold3 to predict the spatial structure of the JASPAR database prediction results and displayed the binding domain with PyMOL (Fig. S2B). Subsequent dual-luciferase reporter gene assay indicated that p-P65 could enhance the activity of the wild-type CXCL17 promoter, and this stimulatory effect was largely dependent on the integrity of specific binding sites within the promoter region. (Fig. 7K). In addition, ChIP-qPCR analysis showed a strong binding affinity between p-P65 and the CXCL17 promoter (Fig. 7L). To confirm the regulatory effect of p-P65 on CXCL17 at the transcriptional and translational levels, we performed supplementary qRT-PCR and ELISA assays using stable cell lines. The qRT-PCR results demonstrated that CXCL17 mRNA expression was significantly upregulated in the TNF-α treated group and SNRPC overexpression stable cell line, while markedly downregulated in the IKK16 treated group and SNRPC shRNA stable cell line. Consistent with the mRNA changes, ELISA assays showed that secreted CXCL17 protein levels exhibited the same expression pattern. Together with the dual-luciferase reporter assay data, these results confirm that p-P65 transcriptionally activates CXCL17 expression by binding to its promoter. These findings establish a complete regulatory cascade: SNRPC activates the NF-κB pathway, increases p-P65 levels, enhances CXCL17 promoter activity, and ultimately upregulates CXCL17 at both mRNA and protein levels (Figs. S2C, D).

### WT cells release CXCL17 into the tumor microenvironment partially dependent on the migrasome pathway

Migrasomes are newly discovered vesicles formed on retraction fibers of migrating cells, mediating migratory exocytosis and defined as a novel membranous organelle. Migrasomes form during cell migration and are involved in intercellular communication, homeostasis maintenance, embryonic development, and the pathogenesis, progression, and diagnosis of various diseases. Studies have shown that migrasomes are loaded with a large number of signaling molecules such as chemokines, cytokines, and growth factors, which may imply that they have very complex and specific intercellular communication functions. In this study (Fig. S3A), transcriptome sequencing data showed that the expression of migrasome marker proteins TSPAN4 and TSPAN7 was significantly downregulated in the SNRPC gene knockdown group (Fig. S3B). WGA cell staining results suggested that overexpression of the SNRPC gene significantly promoted migrasome formation in the two WT cell lines, and subsequent knockdown of the key migrasome formation protein TSPAN4 in the SNRPC gene overexpression cell line partially inhibited migrasome formation (Fig. S3C). ELISA results suggested that the concentration of CXCL17 was significantly decreased after TSPAN4 knockdown in the culture supernatants of the two cell lines (Fig. S3D). In addition, flow cytometry showed that TSPAN4 knockdown could partially reduce the M2-type TAM polarization rate caused by SNRPC gene overexpression (Fig. S3E). These results collectively suggest that WT cells may have a potential regulatory mechanism of releasing CXCL17 through the migrasome pathway to drive M2-type TAM infiltration in WT and induce chemoresistance.

### Preparation, characterization, in vitro uptake, and therapeutic efficacy of hybrid exosomes (hEVs)

**Fig. 8:**
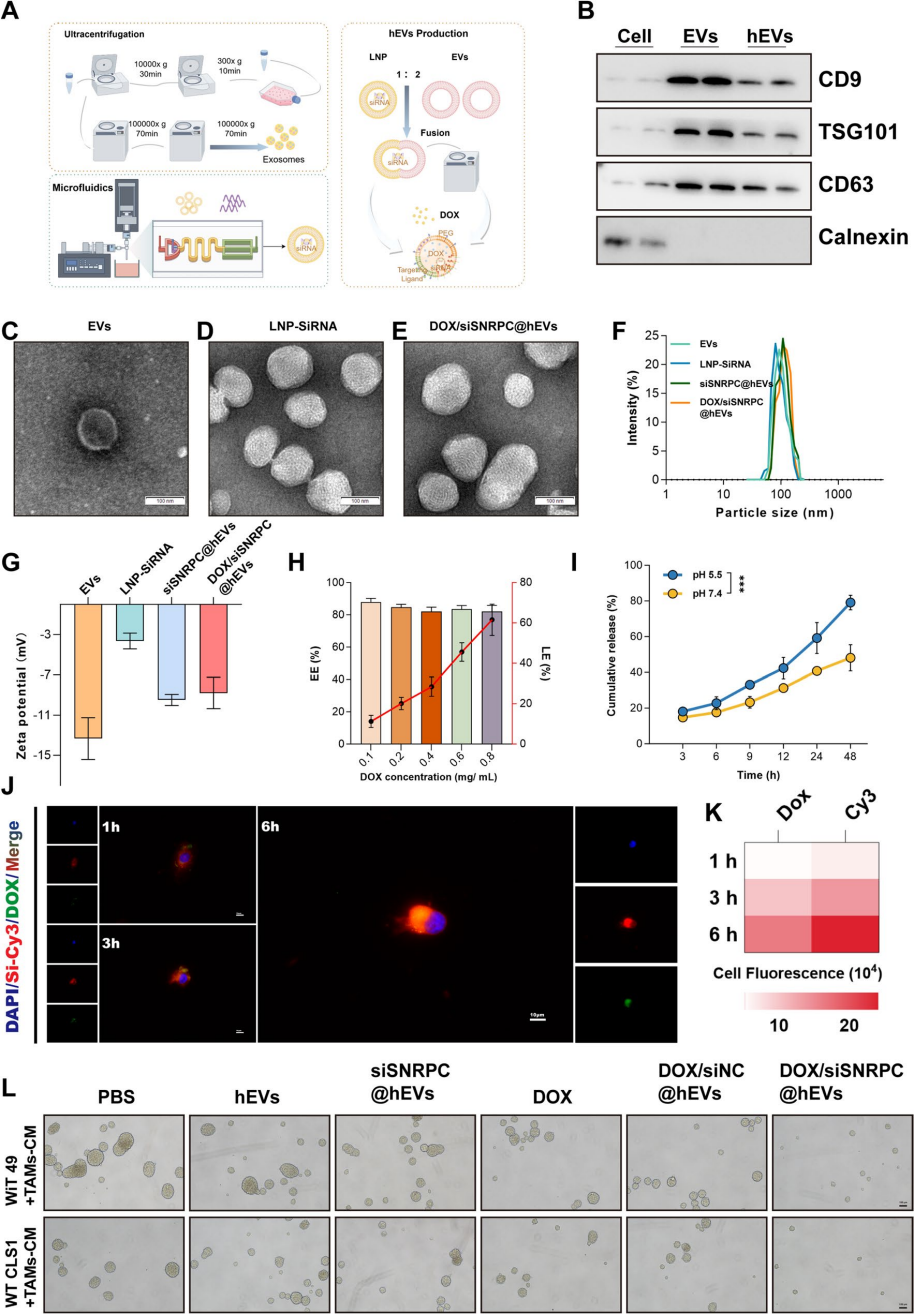
Construction and characterization of hybrid exosomes. **A** Schematic diagram showing the construction process of EVs, LNP-siRNA, and hybrid exosomes. **B** WB analysis of CD9, CD63, TSG101, and Calnexin expression in EVs and hybrid exosomes. **C**-**E** Transmission electron microscopy (TEM) images of EVs, LNP-siRNA, and DOX/siSNRPC@hEVs. **F** Nanoparticle tracking analysis (NTA) showing the size distribution and concentration of EVs, LNP-siRNA, siSNRPC@hEVs, and DOX/siSNRPC@hEVs. **G** Zeta potential of EVs, LNP-siRNA, siSNRPC@hEVs, and DOX/siSNRPC@hEVs. **H** Encapsulation efficiency (EE) and loading efficiency (LE) of DOX in DOX/siSNRPC@hEVs. **I** Drug release efficiency of DOX/siSNRPC@hEVs under pH 5.5 and 7.4 conditions. **J**, **K** Uptake of DOX/siSNRPC@hEVs by WiT49 cells at different time points after intervention. **L** Volume changes of 3D spheroids formed by WiT49/WT-CLS1 cells and PMA-treated THP-1 cells after different drug interventions

First, we extracted exosomes secreted by bone marrow mesenchymal stem cells (BMSCs) and fused them with liposome membranes produced by a microfluidic device to prepare hybrid exosomes (hEVs), then prepared DOX-loaded hybrid exosomes (hEVs) using the ammonium sulfate gradient method (Fig. 8A). Transmission electron microscopy showed the morphology of EVs, LNP-siRNA, and DOX/siSNRPC@hEVs, all displaying bilayer membrane structures (Figs. 8C-E). Nanoparticle tracking analysis indicated that the peak diameter of DOX/siSNRPC@hEVs was 149.3 ± 12.1 nm (Fig. 8F), similar in size to EVs, LNP-siRNA, and siSNRPC@hEVs. Western blot analysis showed that hEVs expressed exosome markers CD9, CD63, and tumor susceptibility gene 101 (TSG101), but not the negative marker calnexin (Fig. 8B), indicating successful fusion of liposomes and exosomes. In addition, we measured the surface charge of EVs, LNP-siRNA, siSNRPC@hEVs, and DOX/siSNRPC@hEVs (Fig. 8G). We studied the drug loading and release profiles of DOX/siSNRPC@hEVs. Results showed that even at a DOX concentration of 0.8 mg/mL, the drug encapsulation efficiency was high (approximately 80%), while the loading efficiency continued to increase and could reach approximately 60% (Fig. 8H). In addition, we also studied the drug release profiles of DOX/siSNRPC@hEVs under simulated physiological conditions (pH 7.4) and the internal environment of intratumoral lysosomal compartments (pH 5.5). As shown in Fig. 8I, DOX/siSNRPC@hEVs were rapidly released in an acidic environment; however, their release rate was slower under physiological conditions.

To detect the in vitro uptake of DOX/siSNRPC@hEVs, we synthesized DOX/siSNRPC@hEVs using CY3 fluorescently labeled siRNA, then monitored the cell uptake using WiT49 cells. Utilizing the characteristic of DOX autofluorescence, we found that DOX/siSNRPC@hEVs were effectively internalized into cells with increasing incubation time (Figs. 8J, K). In addition, 3D spheroids constructed using the two WT cell lines were co-cultured with TAMs in a Transwell system. Under the same dose of DOX intervention, DOX/siSNRPC@hEVs exerted a significant tumor inhibitory effect, and its therapeutic effect was superior to that of the DOX alone and siRNA groups (Fig. 8L).

### DOX/siSNRPC@hEVs exert significant antitumor efficacy via the SNRPC-NF-κB-CXCL17-M2-type TAMs signaling axis in orthotopic tumor models and show excellent tumor targeting

**Fig. 9:**
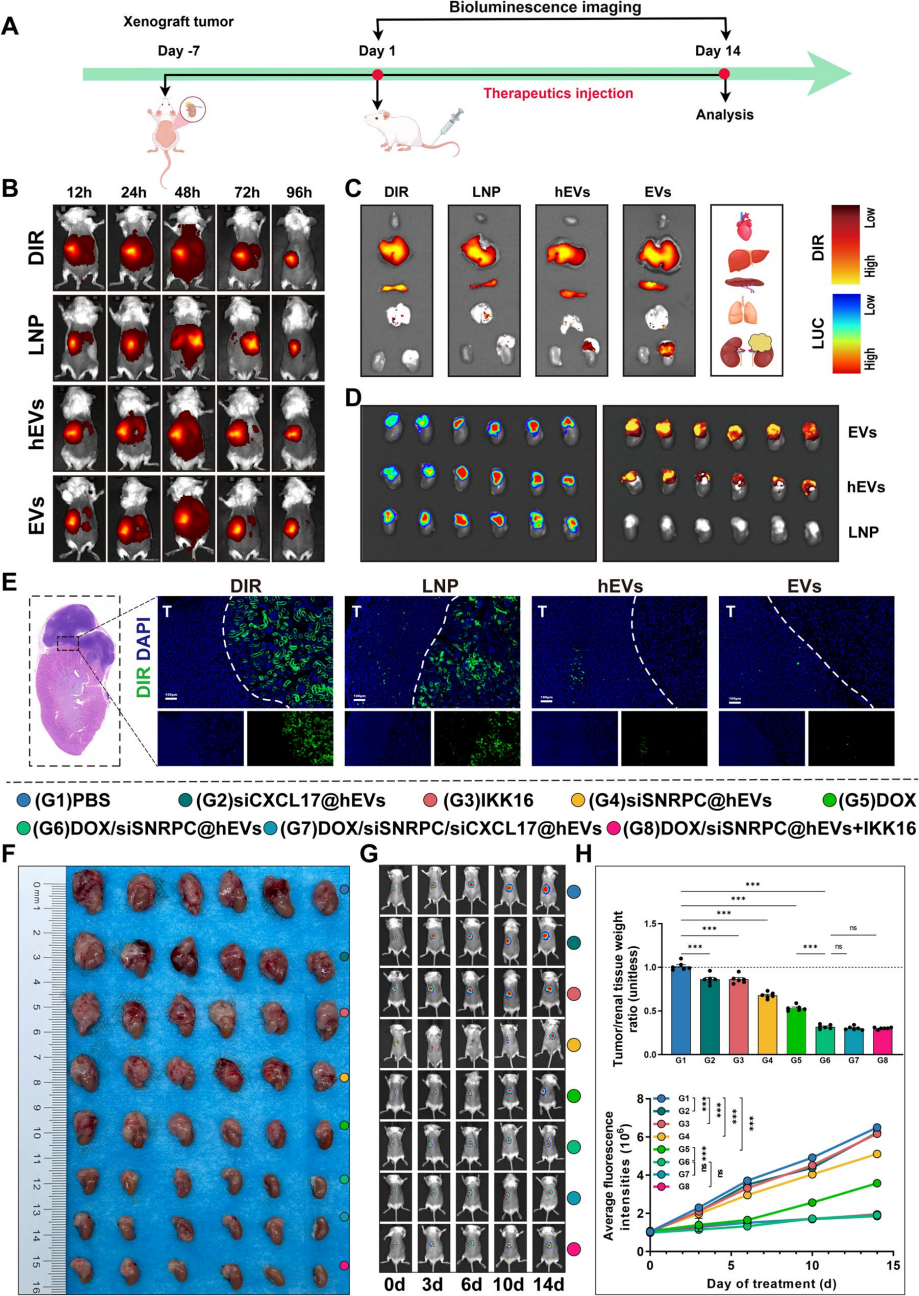
Targeting ability of hybrid exosomes and in vivo efficacy of drug-loaded hybrid exosomes. **A** Schematic diagram of the animal experiment. **B** In vivo imaging showing the distribution of free DIR, EVs, LNP, and hEVs at different time points after administration. **C** Organ distribution of drugs in each group at 48 h after administration. **D** Drug distribution in orthotopic tumors of each group at 48 h after administration. **E** Frozen section analysis showing the distribution of free DIR, EVs, LNP, and hEVs in tumors and kidneys. **F**-**H** In vivo imaging monitoring and statistical analysis of fluorescence intensity changes in orthotopic tumors under different drug interventions and representative images and relative volume comparison of orthotopic tumors after different interventions

To evaluate the therapeutic potential of DOX/siSNRPC@hEVs, we established an orthotopic renal tumor model co-transplanted with SNRPC-overexpressing WiT49 and TAMs (Fig. 9A). First, the tumor targeting ability of DOX/siSNRPC@hEVs was tested. Free DIR solution, as well as LNP, hEVs, and EVs labeled with the same concentration of DIR, were injected via the tail vein respectively. In vivo imaging results of mice showed that the liver was the main organ with fluorescent accumulation in several groups of drugs, and the concentration peaked at 48 hours after administration (Fig. 9B). Considering that the fluorescence of orthotopic renal tumors was masked by the liver and spleen in in vivo imaging, which could not effectively show the real organ distribution, we dissected the organs of mice in each group for detection at 48 hours after administration. Results found that the fluorescence intensity of the liver and spleen was the strongest (Fig. 9C), significantly higher than that of other organs. EVs had the strongest fluorescence in orthotopic tumors, followed by hEVs, while LNP was not detected in orthotopic tumors (Fig. 9D). DIR immunofluorescence detection of orthotopic tumors also verified the same conclusion (Fig. 9E). These results indicate that hEVs, generated by hybridization of LNP and EVs, possess the tumor targeting ability of EVs and can effectively target tumors in vivo.

Subsequently, in vivo imaging and gross orthotopic tumor results of the 14-day administration in vivo treatment model suggested that inhibiting the SNRPC gene, NF-κB activation, and CXCL17 gene alone in the SNRPC-NF-κB-CXCL17-M2-type TAMs signaling axis could significantly inhibit tumor growth. In addition, compared with the single use of siCXCL17, IKK16, or DOX, DOX/siSNRPC@hEVs significantly reduced tumor volume, effectively inhibited tumor cell proliferation, and achieved excellent antitumor efficacy, while combined use with siCXCL17 and IKK16 did not show a more significant tumor inhibitory effect (Figs. 9F-H). Furthermore, ELISA was performed on mouse tumor tissues to determine the CXCL17 content, which demonstrated that DOX/siSNRPC@hEVs could effectively inhibit the production of CXCL17 in tumor tissues(Fig. S4A). Similarly, immunofluorescence staining of M2-TAMs in tumor tissue sections revealed that DOX/siSNRPC@hEVs could effectively suppress the polarization of M2-TAMs in tumor tissues(Fig. S4B), thereby achieving the goal of inhibiting tumor growth.

In addition, we designed another group of in vivo experiments to verify the effectiveness and biosafety of the constructed nanomedicine. In vivo imaging and gross orthotopic tumor results of the 14-day administration in vivo treatment model suggested that hEVs and siNC had no antitumor effect, excluding the influence of the drug carrier, and DOX/siSNRPC@hEVs could exert the best antitumor efficacy (Figs. S5A, B). In addition, we compared the pathological results of the heart, liver, spleen, lung, and kidney of mice treated with DOX/siSNRPC@hEVs and the control group, cardiac fibrosis (Masson staining), and blood toxicity of mice (liver function, renal function, and myocardial enzymes). Results suggested more severe organ damage in the free doxorubicin group, especially significant cardiac damage indicated by Masson staining of the heart. In addition, ALT, AST, CREA, BUN, LDH, and CK in the free doxorubicin group were significantly higher than those in other groups, but the DOX/siSNRPC@hEVs group did not show obvious toxicity (Figs. S5C, D). This suggests that DOX/siSNRPC@hEVs can exert a strong targeting effect on the premise of effective therapeutic effect, avoiding systemic toxicity, especially effectively avoiding DOX-induced cardiotoxicity and protecting the cardiac function of mice. These in vivo efficacy results indicate that DOX/siSNRPC@hEVs are effective and safe for the treatment of WT.

### DOX/siSNRPC@hEVs show excellent tumor targeting and significant antitumor efficacy in lung metastasis tumor models

**Fig. 10:**
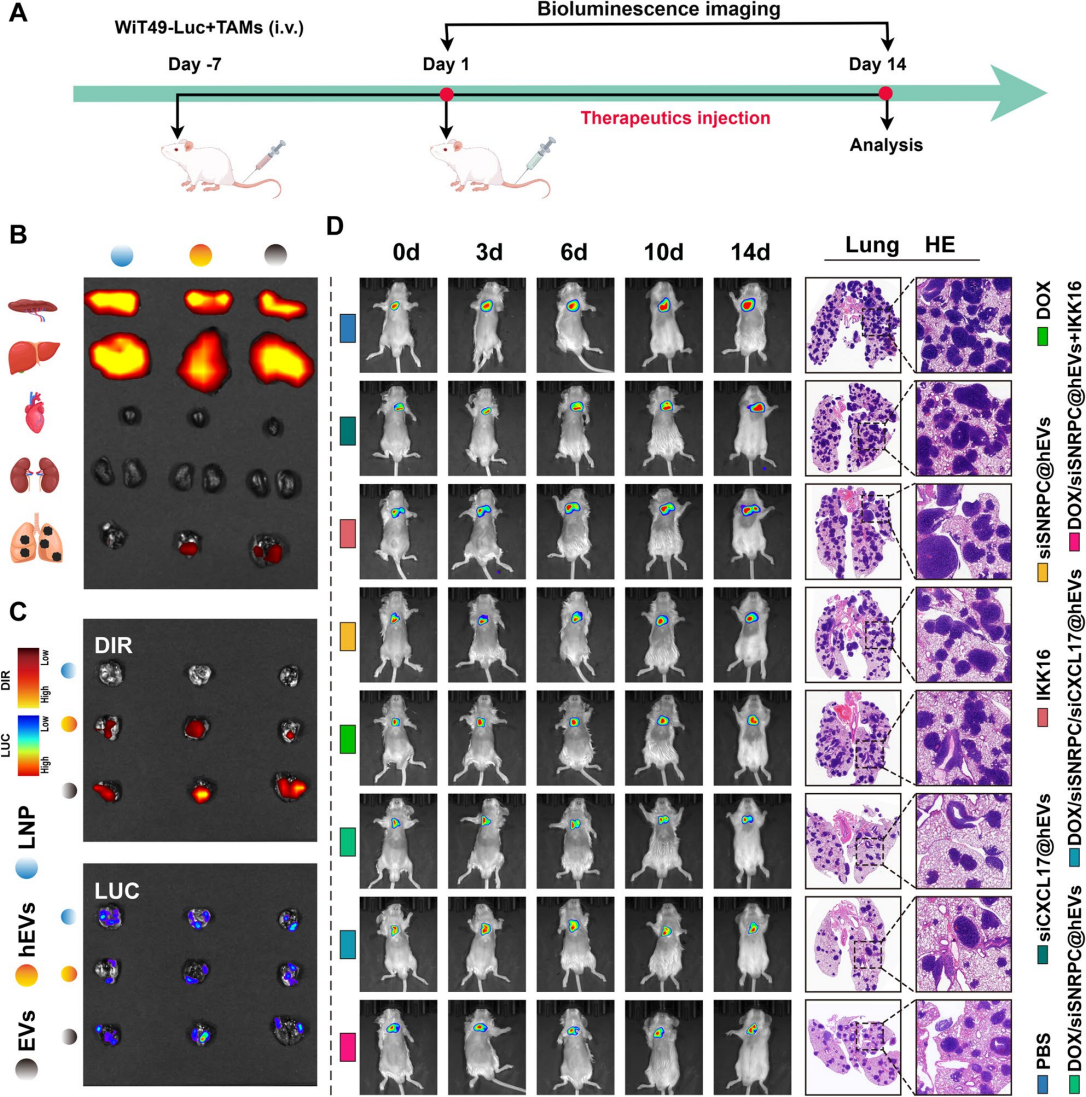
Targeting ability of hybrid exosomes to lung metastases and in vivo efficacy of drug-loaded hybrid exosomes. **A** Schematic diagram of the animal experiment. **B** Organ distribution of drugs in each group at 48 h after administration. **C** Drug distribution in orthotopic tumors of each group at 48 h after administration. **D** In vivo imaging monitoring fluorescence changes in lung metastases and HE staining showing the number and size of lung metastases in each group

The lung is the most common metastatic organ of Wilms tumor, and once lung metastasis occurs, the prognosis is often poor. Therefore, how to effectively treat WT lung metastases is a very difficult but important issue in WT treatment. Therefore, we established a WT lung metastasis model to evaluate whether DOX/siSNRPC@hEVs can target lung metastases and effectively inhibit lung metastases (Fig. 10A). Similar to the orthotopic tumor study, we first evaluated the targeting of the drug using ex vivo organ fluorescence imaging, then evaluated the antitumor efficacy of the drug using in vivo imaging and gross tumors after the intervention cycle (Figs. 10B, C). Results suggested that EVs and hEVs also showed good targeting to lung metastases. In addition, to more accurately evaluate the antitumor efficacy of six different drug interventions, we used in vivo imaging, gross lung evaluation, and panoramic HE staining of the lung to assess the number and size of lung metastatic lesions (Fig. 10D). Results showed that DOX/siSNRPC@hEVs effectively inhibited the growth of lung metastatic lesions and also achieved excellent antitumor effect on metastatic lesions. Combined with the intervention results of orthotopic tumors and lung metastases, we found that DOX/siSNRPC@hEVs showed good targeting, safety, and excellent antitumor efficacy in both orthotopic tumors and lung metastases.

## Discussion

Wilms tumor, the most common malignant renal tumor in children, currently achieves curative effects in most patients with the combination of surgery, chemotherapy, and radiotherapy. However, the recurrence rate remains significantly high in high-risk subtypes such as blastemal-predominant WT and diffuse anaplastic WT, with chemoresistance being the core issue leading to treatment failure [2, 3, 26]. Aberrant remodeling of the TME plays a crucial role in WT progression and drug resistance [9, 10, 27]. Among them, the infiltration pattern of immune cells is closely related to prognosis [28, 29]. This study focused on the M2-type TAMs-mediated resistance mechanism in TME, revealed a novel mechanism by which SNRPC promotes WT chemoresistance through regulating the immune microenvironment, and developed a targeted nanotherapeutic system, providing experimental evidence for improving the prognosis of WT.

In the TME of WT, M2-type TAMs, as the most abundant immune cell population, create an immunosuppressive environment by secreting anti-inflammatory factors such as IL-10 and TGF-β, and remodel vascular and stromal structures by releasing VEGF and CXCL family chemokines, thus providing a sanctuary for tumor cells [30–33]. Clinical sample analysis in this study showed that the proportion of M2-type TAMs (CD68⁺CD163⁺) in WT tissues from the chemoresistant group was significantly higher than that in the non-chemoresistant group, which was closely associated with reduced long-term survival rate, confirming the clinical correlation between M2-type TAMs infiltration and WT chemoresistance. Further WGCNA combined with prognostic data identified SNRPC as a key gene associated with M2-type TAMs infiltration. Clinical validation revealed that SNRPC was highly expressed in WT tumor tissues, especially in the chemoresistant group, and was positively correlated with the M2-type TAMs marker CD163, serving as an independent risk factor for poor prognosis in WT patients. Functional experiments demonstrated that SNRPC not only promotes malignant phenotypes of WT cells such as proliferation, migration, and invasion, but also induces TAM-dependent chemoresistance by regulating TME—SNRPC knockdown significantly reduced the polarization rate of M2-type TAMs, while overexpression promoted their infiltration. Importantly, this regulation does not depend on direct effects on the intrinsic drug resistance of tumor cells, suggesting that SNRPC may be involved in drug resistance through regulating the crosstalk between tumor and immune cells.

SNRPC is a core component of the spliceosomal small nuclear ribonucleoprotein complex, traditionally recognized for its role in pre-mRNA splicing and RNA processing [34]. Emerging evidence suggests that SNRPC dysregulation contributes to tumorigenesis beyond its canonical functions. For instance, SNRPC overexpression has been linked to enhanced cell proliferation and metastasis in several cancers, including hepatocellular carcinoma and breast cancer, by modulating oncogenic signaling pathways [35, 36]. However, its role in regulating the immune microenvironment and chemoresistance remained unexplored prior to this study. Here, we identified SNRPC as a critical bridge between tumor cells and M2-type TAMs in WT: its high expression in chemoresistant WT tissues correlates with M2-type TAMs infiltration and predicts poor prognosis, highlighting a novel non-cell-autonomous role of SNRPC in tumor-immune crosstalk. This expands SNRPC’s known functions from intracellular RNA metabolism to extracellular microenvironment regulation, providing a new perspective for understanding its oncogenic potential.

To clarify the molecular mechanism by which SNRPC regulates M2-type TAMs infiltration, transcriptome sequencing in this study found that SNRPC knockdown significantly inhibited the activation of the NF-κB signaling pathway. Further mechanistic studies confirmed that SNRPC promotes the nuclear translocation of NF-κB p65 by activating the IKKα/β-IκBα-p65 cascade, and the translocated p65 can directly bind to the promoter of CXCL17 and upregulate its expression. Chemokines mediate immune cell trafficking and TME remodeling by recruiting pro-tumor or anti-tumor immune subsets [37]. CXCL17, a less-studied chemokine with chemotactic activity for monocytes, macrophages, and neutrophils, promotes tumor progression in cancers like gastric and lung cancer by fostering immunosuppressive microenvironments [38–40]. In this study, we found CXCL17 promotes M2-type TAMs polarization in a dose-dependent manner in WT, and exogenous supplementation of CXCL17 can partially reverse the inhibitory effect of SNRPC knockdown on M2-type TAMs infiltration, indicating that CXCL17 is a key effector molecule in the regulation of TME by the SNRPC-NF-κB axis. Notably, this study also found that WT cells can release CXCL17 through a migrasome-dependent pathway: SNRPC knockdown significantly reduced the expression of migrasome markers TSPAN4/7 and migrasome formation, while TSPAN4 knockdown decreased the concentration of CXCL17 in cell supernatants, suggesting that SNRPC may enhance M2-type TAMs infiltration by regulating migrasome-mediated CXCL17 release. This finding provides a new perspective for understanding the non-classical pathways of cytokine secretion.

Strategies delivering RNA molecules (siRNA, miRNA, shRNA) to inhibit abnormal gene expression show promise in disease and cancer treatment, with RNAi technology enabling oncogene inactivation or suppression of cell migration/proliferation via exogenous small RNAs [41–44]. However, siRNA’s large molecular weight (~ 13–14 kDa) and highly negatively charged phosphate backbone hinder membrane penetration, and it is prone to RNase degradation, necessitating better delivery carriers despite stability-enhancing chemical modifications [20]. Targeted drug delivery, which improves efficacy by precise drug localization to reduce doses and side effects, has seen EVs—naturally occurring nanovesicles with good biocompatibility, dual transport of hydrophilic/hydrophobic molecules, and tissue targeting—gain attention [45, 46]. Yet EVs suffer from low siRNA encapsulation efficiency, which is addressed by membrane fusion with microfluidic-prepared siRNA liposomes (mature, stable, high encapsulation) [23, 47, 48]. This hybrid nanovesicle retains EV-mediated targeting/biocompatibility and liposome-based high siRNA loading, optimizing drug loading and targeted delivery.

For targeting the TME resistance mechanism regulated by SNRPC, this study designed a hEVs nano-delivery system, which fuses BMSCs-derived exosomes with liposomes to co-deliver DOX and siSNRPC. Characterization results showed that DOX/siSNRPC@hEVs had a uniform particle size distribution (approximately 149 nm) and high encapsulation efficiency (DOX encapsulation efficiency of approximately 80%), and accelerated drug release in an acidic environment (pH 5.5), adapting to the pH characteristics of the tumor microenvironment. In vitro experiments confirmed that hEVs can be efficiently internalized by WT cells, and DOX/siSNRPC@hEVs more significantly inhibited tumor cell proliferation and M2-type TAMs polarization compared with free DOX or siRNA alone, showing stronger tumor-killing efficacy in 3D spheroid models. In vivo studies further verified the advantages of this nano-system: in the orthotopic WT model, DOX/siSNRPC@hEVs significantly improved tumor targeting, reduced liver and spleen accumulation, and more effectively reduced tumor volume and M2-type TAMs infiltration compared with free DOX; in the lung metastasis model, it effectively inhibited the growth of metastatic lesions and prolonged the survival of mice. More importantly, this system significantly reduced DOX-induced cardiotoxicity and hepatorenal function damage, addressing the clinical issue of severe side effects of traditional chemotherapeutic drugs. Notably, DOX/siSNRPC@hEVs showed preferential accumulation in the liver compared to the lungs (Fig. 9B). This observation raises the possibility that the nanosystem may exert enhanced therapeutic effects against liver metastases of WT, which are clinically relevant in high-risk patients. Future studies will establish orthotopic WT models with liver metastasis to directly validate this hypothesis, evaluating tumor burden, metastatic nodule formation, and survival outcomes. Additionally, we will explore the underlying mechanism of liver targeting, including the interaction between hEV surface markers and liver sinusoidal endothelial cells, to further optimize the delivery efficiency for liver-localized metastases.

For the treatment of lung metastases, the relatively lower lung accumulation of DOX/siSNRPC@hEVs suggests room for optimization. We plan to modify the hEV surface with lung-homing ligands to improve tissue-specific targeting—for example, conjugating RGD peptides (which bind to integrins highly expressed on lung metastatic tumor cells) or CXC chemokine receptor ligands (which recognize receptors on lung endothelial cells). In vitro transwell assays and in vivo lung metastasis models will be used to verify whether modified hEVs exhibit increased lung accumulation and enhanced antitumor efficacy. Moreover, we will adjust the dosage and administration route (e.g., aerosol inhalation) to further improve the bioavailability of the nanosystem in the lungs.

Admittedly, this study still has certain limitations: the clinical sample size is limited, and the correlation between SNRPC and M2-type TAMs infiltration as well as their prognostic value need to be further verified in multi-center large-cohort studies; the specific molecular mechanism of migrasome-mediated CXCL17 release remains to be fully elucidated; the large-scale preparation process, long-term safety, and efficacy of the hEVs nano-system in PDX models require systematic evaluation. Nevertheless, the SNRPC-NF-κB-CXCL17-M2 TAMs regulatory axis revealed in this study provides new insights into understanding the molecular mechanism of WT chemoresistance, and the developed DOX/siSNRPC@hEVs nano-system confirms the feasibility of targeted TME regulation combined with chemotherapy, offering new experimental evidence and potential strategies for improving the therapeutic efficacy of high-risk WT.

## Conclusion

In summary, this study clarifies the chemoresistance regulatory mechanism in WT and develops a targeted nanotherapeutic strategy. Clinical and experimental evidence confirms that elevated infiltration of M2-type tumor-associated macrophages (TAMs, CD68⁺CD163⁺) in high-risk WT is closely associated with chemoresistance and poor prognosis, being a key mediator of treatment resistance. SNRPC is identified as a critical microenvironmental regulator: highly expressed in chemoresistant tissues, it promotes tumor cell malignancy and drives M2-type TAM infiltration/polarization via non-cell-autonomous effects to induce TAM-dependent chemoresistance, with its expression correlating positively with M2-type TAM levels and serving as an independent prognostic risk factor. Mechanistically, SNRPC activates the NF-κB pathway by promoting IKKα/β and IκBα phosphorylation, inducing NF-κB p65 nuclear translocation to upregulate CXCL17, which then recruits and polarizes M2-type TAMs in a dose-dependent manner; SNRPC also modulates migrasome-dependent CXCL17 release via regulating TSPAN4. Finally, the hybrid exosome nanosystem (DOX/siSNRPC@hEVs) efficiently co-delivers doxorubicin and siSNRPC to tumors, enhancing targeting, reducing systemic toxicity (especially cardiotoxicity), inhibiting orthotopic tumor growth and lung metastases, and reversing chemoresistance by reducing M2-type TAM infiltration. These findings highlight the critical role of the SNRPC-NF-κB-CXCL17-M2 TAMs axis in WT chemoresistance and validate DOX/siSNRPC@hEVs’ therapeutic potential, providing new biomarkers and targeted strategies for high-risk WT.

## Supplementary Information

Supplementary Material 1.

Supplementary Material 2.

Supplementary Material 3.

Supplementary Material 4: Fig. S1. Screening of Potential Chemoresistance-Related Genes Associated with Macrophage Polarization in WT via Immunofluorescence Staining and Flow Cytometry. A. Immunofluorescence staining was performed to detect the expression levels of PSMA4, PPIH, PFDN4 and CKS1B in clinical tumor tissues, adjacent non-tumor tissues, as well as chemoresistant and chemosensitive clinical tissues, respectively. B. Flow cytometry was used to detect the effects of SNRPC, PSMA4, PPIH, PFDN4 and CKS1B knockdown on M2 polarization of macrophages under co-culture conditions, respectively. Fig. S2. Predicted structural model of p-P65 binding to the CXCL17 promoter using AlphaFold3. A. Predicted binding sites of p-P65 to the CXCL17 promoter using the JASPER database. B. The spatial structure of the potential binding sites between p-P65 and the CXCL17 promoter predicted by AlphaFold3 was visualized using PyMOL. C. Relative CXCL17 mRNA levels in control, TNF-α, IKK16, SNRPC knockdown, and SNRPC overexpression stable cell lines. D. Secreted CXCL17 protein levels in cell supernatants from the indicated groups. Fig. S3. Migrasome detection assays. A. Flow Chart of the Research Procedure. B. Expression changes of migrasome-related genes TSPAN4, TSPAN7, and TSPAN9 after SNRPC knockdown. C. WGA staining showing migrasome formation in WiT49/WT-CLS1 cells with SNRPC overexpression (oe_SNRPC) or silencing. D. ELISA analysis of CXCL17 concentration in cell supernatants after TSPAN4 knockdown in WiT49/WT-CLS1 cells. E. Flow cytometry analysis of CD68⁺CD206⁺ macrophage proportion in the Transwell co-culture system, with TSPAN4-silenced WiT49/WT-CLS1 cells in the upper chamber and PMA-treated THP-1 cells in the lower chamber. Fig. S4. Effects of DOX/siSNRPC@hEVs on CXCL17 Secretion and M2-TAM Polarization in Tumor Tissues. A. ELISA was performed to detect the content of CXCL17 in mouse tumor tissues; B. Immunofluorescence staining was conducted to observe the polarization of M2-TAMs in tumor tissue sections. Fig. S5. In Vivo Evaluation of Biosafety and Efficacy of DOX/siSNRPC@hEVs. A-B. Representative images and relative volume comparison of orthotopic tumors after different interventions. C. HE staining analyzing pathological damage in the heart, liver, spleen, lung, and kidney, and Masson staining detecting cardiac fibrosis in each group. D. Heatmap showing the levels of liver function indices, renal function indices, and myocardial enzymes in each group.

## Data Availability

Data produced in this study are available within the article and its accompanying supplementary files.

## Abbreviations

BMSCs: bone marrow mesenchymal stem cell
CXCL17: C-X-C motif chemokine ligand 17
CCK-8: Cell Counting Kit-8
DOX: doxorubicin
EVs: extracellular vesicles
EE: encapsulation efficiency
GSEA: gene set enrichment analysis
hEVs: hybrid exosomes
IF: Immunofluorescence
LE: Loading efficiency
NF-κB: nuclear factor-κB
OS: overall survival
PDX: patient-derived xenograft models
qRT-PCR: Quantitative Real-Time Polymerase Chain Reaction
SNRPC: small nuclear ribonucleoprotein polypeptide C
siRNA: Small interfering RNA
TME: Tumor microenvironment
TAMs: tumor-associated macrophages
TIICs: tumor-infiltrating immune cells
WT: Wilms tumor
WGCNA: Weighted gene co-expression network analysis
WB: Western Blot
