# Supplementary material for: SNRPC promotes chemoresistance in Wilms tumor via the NF-κB-CXCL17 axis regulating M2-Type TAMs infiltration and targeted nanotherapy research
Source: J Exp Clin Cancer Res. 2026 Feb 28;45:97. doi: 10.1186/s13046-026-03680-z (PMC13067748; doi:10.1186/s13046-026-03680-z)
Supplement: Supplementary file 1 — Supplementary Material 1. [file 13046_2026_3680_MOESM1_ESM.pdf]

# 重庆医科大学附属儿童医院
# 动物实验伦理审查批准书

批准文号（IACUC Issue No）：CHCMU-IACUC20250429002

本《动物实验方案》经过实验动物福利伦理审查委员会审核，符合动物保护、动物福利和伦理原则，符合国家实验动物伦理福利的相关规定。

The animal use protocol has been reviewed and approved by the Ethics Committee of Children's Hospital of ChongQing Medical University.

<table>
<tr><td rowspan="2">课题名称<br>Project Title</td><td colspan="5">肿瘤相关巨噬细胞对肾母细胞瘤化疗耐药的影响及其机制研究</td></tr>
<tr><td colspan="5">The Impact of Tumor-Associated Macrophages on Chemotherapy Resistance in Wilms' Tumor and Its Mechanisms</td></tr>
<tr><td colspan="2" rowspan="2">课题负责人<br>Principle Investigator (PI)</td><td colspan="2">何大维</td><td rowspan="2">科室<br>Department</td><td>泌尿外科</td></tr>
<tr><td colspan="2">He Dawei</td><td>Department of Urology</td></tr>
<tr><td rowspan="2">申请人<br>Applicant</td><td colspan="2">孔祥盼</td><td colspan="2">申请日期<br>Application date</td><td>2025年3月25日</td></tr>
<tr><td colspan="2">Kong Xiangpan</td><td colspan="2">邮箱<br>Email</td><td>1045189749@qq.com</td></tr>
<tr><td colspan="2">计划执行时间<br>Period of Protocol</td><td colspan="4">2025年5月1日 至 2028年5月1日</td></tr>
<tr><td colspan="2">审查意见<br>Results of inspection</td><td colspan="4">符合动物福利伦理要求，可以开展动物实验 Agree</td></tr>
</table>

签章（实验动物福利伦理审查委员会）
Stamp

日期Date：2025年4月29日
