# Supplementary material for: SNRPC promotes chemoresistance in Wilms tumor via the NF-κB-CXCL17 axis regulating M2-Type TAMs infiltration and targeted nanotherapy research
Source: J Exp Clin Cancer Res. 2026 Feb 28;45:97. doi: 10.1186/s13046-026-03680-z (PMC13067748; doi:10.1186/s13046-026-03680-z)
Supplement: Supplementary file 2 — Supplementary Material 2. [file 13046_2026_3680_MOESM2_ESM.pdf]

# 重庆医科大学附属儿童医院伦理委员会审查批件

## Review and Approval of the Ethics Committee of Children's Hospital Affiliated to Chongqing Medical University

批件号（File No.）：（2025）年伦审（临研）批件第（174）号

| | | | |
|---|---|---|---|
| 项目名称<br>**Protocol name** | 肿瘤相关巨噬细胞对肾母细胞瘤化疗耐药的影响及其机制研究 | | |
| 申办单位<br>**sponsor** | 重庆医科大学附属儿童医院 | | |
| 承担单位<br>**Institute** | 重庆医科大学附属儿童医院 | | |
| 申请科室<br>**Application Department** | 泌尿外科 | 项目负责人<br>**Principal Investigators** | 何大维 |
| 审查日期<br>**Date Reviewed** | 2025.5.21 | 批准日期<br>**Date Approved** | 2025.5.23 |
| 审查文件<br>**Review Document** | 详见提交文件清单（附件） | | |
| 审查方式<br>**Means of Reviewing** | ☐会议审查　会议时间：/<br>☑快速审查　审查时间：2025.4.19、2025.5.21 | | |
| 会议地点<br>**Meetinng Place** | / | | |
| 会议出席委员<br>**Member Present** | / | | |

**审查意见 Decision：**

同意按照批准的文件进行该临床试验。请按照伦理批件上的跟踪审查频率，及时提交年度进展报告；研究结束时，须向本伦理委员会提交结题报告。

**跟踪审查频率 Frequency of Tracking Review：**

☐否　☑是：☐3 个月　☐ 6 个月　☐9 个月　☑12 个月

**批件有效期 Valid Period：2025-5-23 至 2026-5-22**

注意事项（请仔细阅读）：

1. 已批准项目应遵循本伦理委员会批准的方案执行，需符合《涉及人的生命科学和医学研究伦理审查办法》（国卫科教发〔2023〕4 号）、《药物临床试验质量管理规范》（国家药监局、国家卫生健康委令 2020 年第 57 号）、《医疗器械临床试验质量管理规范》（国家食品药品监督管理总局、中华人民共和国国家卫生和计划生育委员会令第 28 号）、WMA《赫尔辛基宣言》和 CIOMS《人体生物医学研究国际道德指南》的伦理原则。
2. 研究过程中若变更主要研究者，对临床研究方案、知情同意书、招募材料等的任何修改，请申请人提交修正案审查申请。
3. 发生严重不良事件，请申请人及时提交严重不良事件报告。
4. 批件有效期为一年，在批准之日起 1 年内启动，逾期未启动的，本批件自行废止，研究未结束需提出延长有效期申请。
5. 请按照伦理委员会规定的跟踪审查频率，在跟踪审查日到期前 1 个月提交研究进展报告。
6. 方案违背/偏离，暂停/提前终止临床研究，需及时通知本伦理委员会。
7. 研究结束时，须向本伦理委员会提交结题报告。

重庆医科大学附属儿童医院医学研究伦理委员会（盖章）
主任/副主任委员：
2025 年 5 月 23 日

申明：本伦理委员会的组成及工作程序符合中国食品药品监督管理局颁布的“药物临床试验质量管理规范”（2020 年）中的相关要求及其所遵循的 ICH GCP 指导原则。

地址：重庆市两江新区金渝大道 20 号
邮编：401122
电话：023-68370035/023-63664659

Address：No20,Jinyu Avenue,Liangjiang New Area,,Chongqing, China.
Tel: 023-68370035/023-63664659
邮箱：chcmull@163.com

地址：重庆市两江新区金渝大道 20 号
邮编：401122
电话：023-68370035/023-63664659

Address：No20,Jinyu Avenue,Liangjiang New Area,,Chongqing, China.
Tel: 023-68370035/023-63664659
邮箱：chcmull@163.com

| 编号 | 文件名称 | 版本/日期 |
| --- | --- | --- |
| 1 | 伦理递交函 | / |
| 2 | 临床立项申请表 | / |
| 3 | 临床研究方案 | 01/2025.03.25 |
| 4 | 研究者利益冲突声明 | / |
| 5 | 知情同意书 | / |
| 6 | 主要研究者履历 | / |
| 7 | 研究团队成员表 | / |
| 8 | 科学性审查立项资料接收函/科学性审查批件 | / |

重庆医科大学附属儿童医院医学研究伦理委员会

| 编号 | 文件名称 | 版本/日期 |
| --- | --- | --- |
| 1 | 复审申请表 | NA |
| 2 | 临床研究方案 | 02/2025. 4. 23 |
| 3 | 研究者履历及伦理证明 | NA |
