# Supplementary material for: SNRPC promotes chemoresistance in Wilms tumor via the NF-κB-CXCL17 axis regulating M2-Type TAMs infiltration and targeted nanotherapy research
Source: J Exp Clin Cancer Res. 2026 Feb 28;45:97. doi: 10.1186/s13046-026-03680-z (PMC13067748; doi:10.1186/s13046-026-03680-z)
Supplement: Supplementary file 3 — Supplementary Material 3. [file 13046_2026_3680_MOESM3_ESM.docx]

**Supplementary Table 1**

| **Targeted Genes** | **Sequence (5′-3′)** |
| --- | --- |
| si-SNRPC (sence) | GGAAUGACUCGACCAGACA(dT)(dT) |
| si-SNRPC(anti-sence) | UGUCUGGUCGAGUCAUUCC(dT)(dT) |
| si-PSMA4 (sence) | GCAGCUGUGUCAAUGUUGAAA |
| si-PSMA4(anti-sence) | UCAACAUUGACACAGCUGCAG |
| si-PPIH (sence) | GCAUGAAGAUCGAGCUCUUUG |
| si-PPIH(anti-sence) | AAGAGCUCGAUCUUCAUGCGG |
| si-PFDN4 (sence) | GCAGAAGAUGUCAAUGUUACU |
| si-PFDN4(anti-sence) | UAACAUUGACAUCUUCUGCAG |
| si-CKS1B(sence) | GACAUGUCAUGCUGCCCAAGG |
| si-CKS1B(anti-sence) | UUGGGCAGCAUGACAUGUCGA |
| si-CXCL17(sence) | GAAUGUGAGUGCAAAGAUU(dT)(dT) |
| si-CXCL17(anti-sence) | AAUCUUUGCACUCACAUUC(dT)(dT) |

**Supplementary Table 2**

| **Name** | **Primer sequence (5’-3’)** | TM (℃) |
| --- | --- | --- |
| CD163 | Forward：ATCAACCCTGCATCTTTAGACA | 60 |
|  | Reverse：CTTGTTGTCACATGTGATCCAG |  |
| CD86 | Forward：TGCTCATCTATACACGGTTACC | 60 |
|  | Reverse：TGCATAACACCATCATACTCGA |  |
| CD68 | Forward：CCCAGATTCAGATTCGAGTCAT | 60 |
|  | Reverse：GTTTTGTTGGGGTTCAGTACAG |  |
| SNRPC | Forward：TGTGACTACTGCGATACATACCT | 60 |
|  | Reverse：GCCTGCTCTTCCATCCATTTCT |  |
| GAPDH | Forward：AAGGTGAAGGTCGGAGTCAAC | 60 |
|  | Reverse：GGGGTCATTGATGGCAACAATA |  |

**Supplementary Table 3**

| **Name** | **No.** | **Primer sequence (5’-3’)** | **bp** |
| --- | --- | --- | --- |
| CXCL17 site1 F | P2509 | TCCATTTGTCTTGGGGGTGT | 131bp |
| CXCL17 site1 R | P2510 | GTTCTTCCAGGTCCTGCTCC |  |
| CXCL17 site2 F | P2511 | GGCTCAGTCATAGCAGTCCC | 158bp |
| CXCL17 site2 R | P2512 | GGCATGAGTCAAAGCCCTTT |  |
| CXCL17 site3 F | P2513 | GGAGCACAGAGGGGAAACAG | 163bp |
| CXCL17 site3 R | P2514 | CTCACACCCCAATGCCTCAT |  |
