# Supplementary material for: SNRPC promotes chemoresistance in Wilms tumor via the NF-κB-CXCL17 axis regulating M2-Type TAMs infiltration and targeted nanotherapy research
Source: J Exp Clin Cancer Res. 2026 Feb 28;45:97. doi: 10.1186/s13046-026-03680-z (PMC13067748; doi:10.1186/s13046-026-03680-z)

**Supplementary Fig.1** Screening of Potential Chemoresistance-Related Genes Associated with Macrophage Polarization in WT via Immunofluorescence Staining and Flow Cytometry. A. Immunofluorescence staining was performed to detect the expression levels of PSMA4, PPIH, PFDN4 and CKS1B in clinical tumor tissues, adjacent non-tumor tissues, as well as chemoresistant and chemosensitive clinical tissues, respectively. **B.** Flow cytometry was used to detect the effects of SNRPC, PSMA4, PPIH, PFDN4 and CKS1B knockdown on M2 polarization of macrophages under co-culture conditions, respectively.

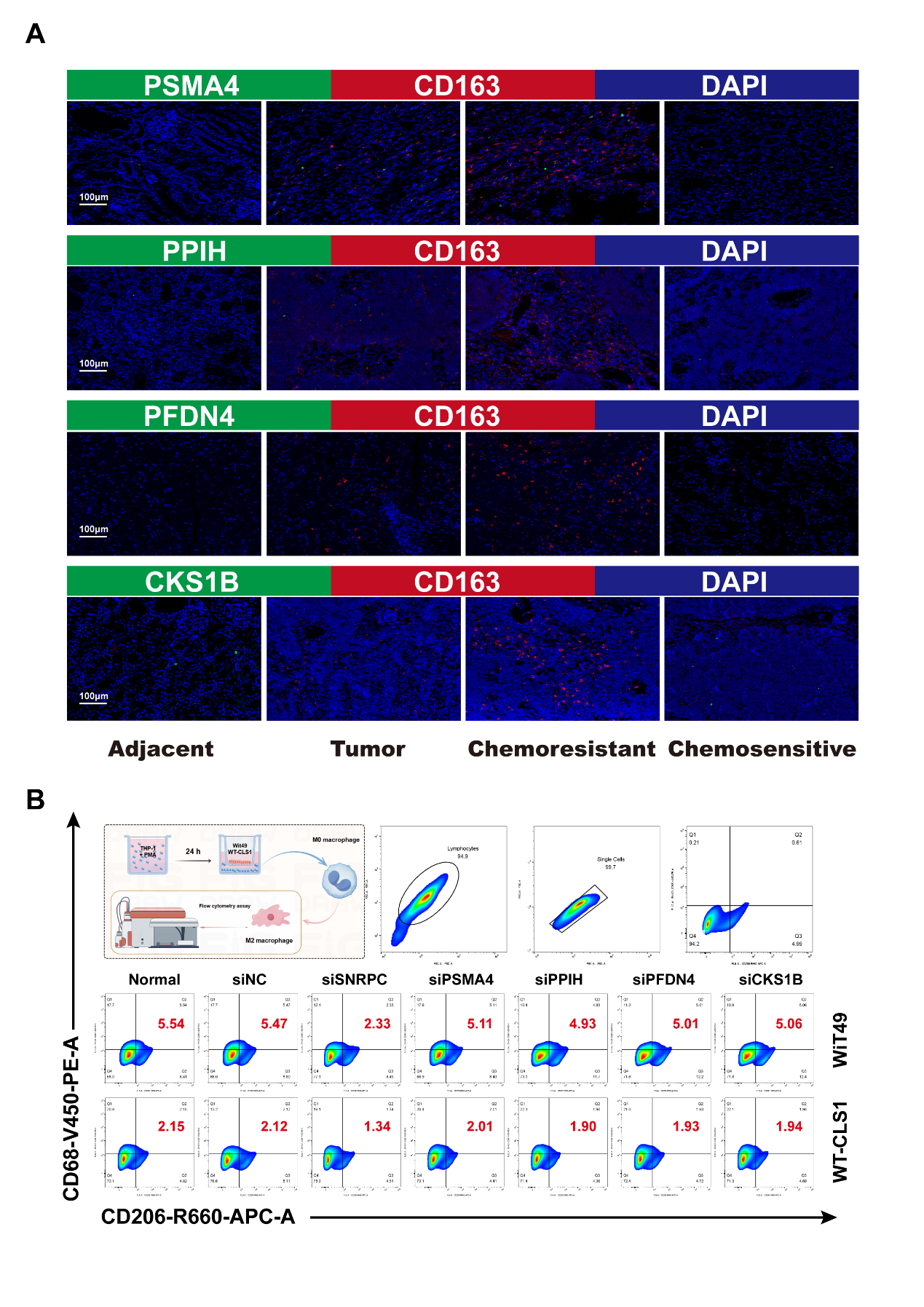


**Supplementary Fig.2** Predicted structural model of p-P65 binding to the CXCL17 promoter using AlphaFold3. **A.** Predicted binding sites of p-P65 to the CXCL17 promoter using the JASPER database. **B.** The spatial structure of the potential binding sites between p-P65 and the CXCL17 promoter predicted by AlphaFold3 was visualized using PyMOL. **C.** Relative CXCL17 mRNA levels in control, TNF-α, IKK16, SNRPC knockdown, and SNRPC overexpression stable cell lines. **D.** Secreted CXCL17 protein levels in cell supernatants from the indicated groups.


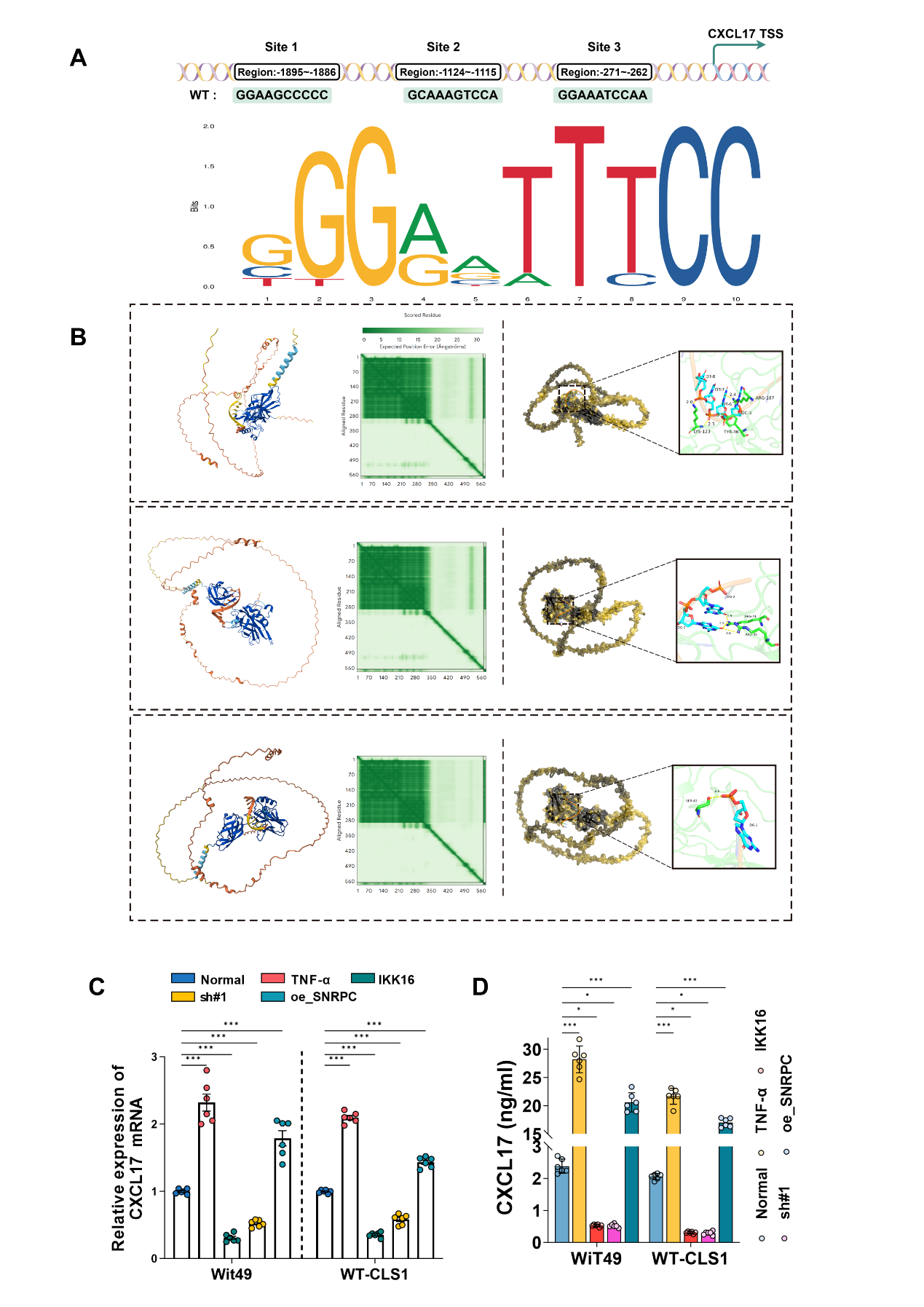


**Supplementary Fig.3** Migrasome detection assays. **A**. Flow Chart of the Research Procedure. **B.** Expression changes of migrasome-related genes TSPAN4, TSPAN7, and TSPAN9 after SNRPC knockdown. **C.** WGA staining showing migrasome formation in WiT49/WT-CLS1 cells with SNRPC overexpression (oe_SNRPC) or silencing. **D.** ELISA analysis of CXCL17 concentration in cell supernatants after TSPAN4 knockdown in WiT49/WT-CLS1 cells. **E**. Flow cytometry analysis of CD68⁺CD206⁺ macrophage proportion in the Transwell co-culture system, with TSPAN4-silenced WiT49/WT-CLS1 cells in the upper chamber and PMA-treated THP-1 cells in the lower chamber.


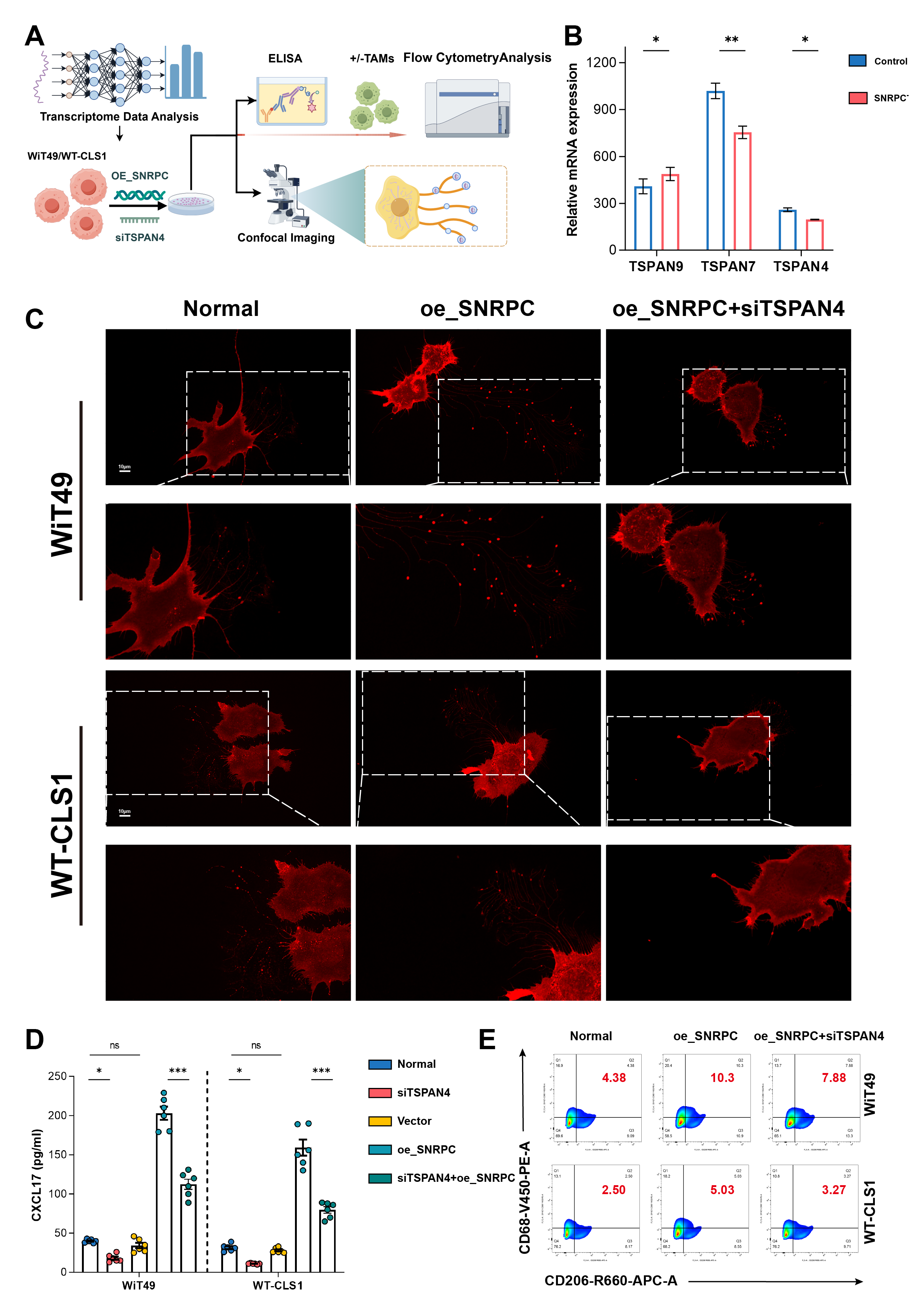


**Supplementary Fig.4** Effects of DOX/siSNRPC@hEVs on CXCL17 Secretion and M2-TAM Polarization in Tumor Tissues. **A.** ELISA was performed to detect the content of CXCL17 in mouse tumor tissues; **B.** Immunofluorescence staining was conducted to observe the polarization of M2-TAMs in tumor tissue sections.


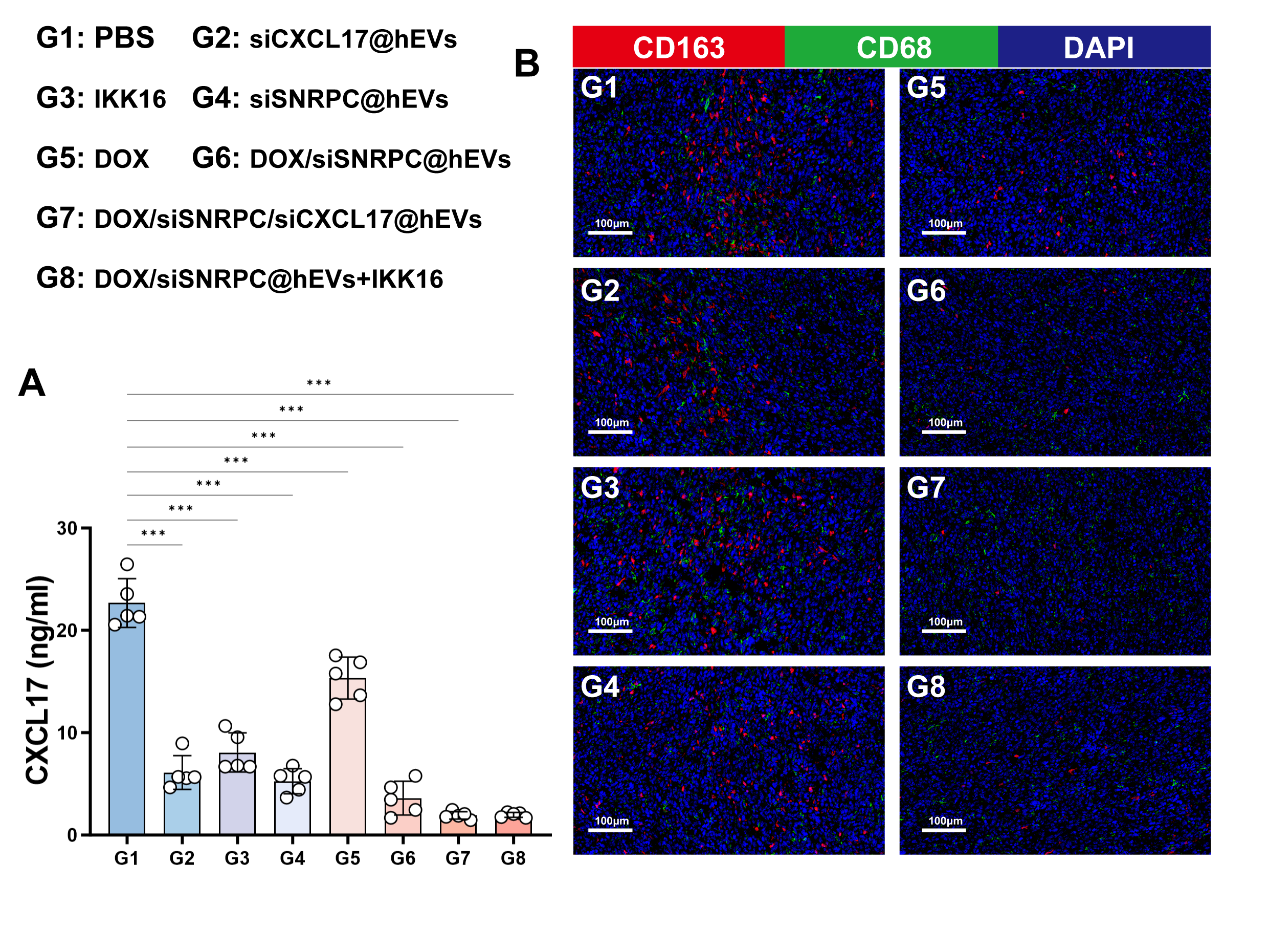


**Supplementary Fig.5** In Vivo Evaluation of Biosafety and Efficacy of DOX/siSNRPC@hEVs. **A-B.** Representative images and relative volume comparison of orthotopic tumors after different interventions. **C.** HE staining analyzing pathological damage in the heart, liver, spleen, lung, and kidney, and Masson staining detecting cardiac fibrosis in each group. **D**. Heatmap showing the levels of liver function indices, renal function indices, and myocardial enzymes in each group.


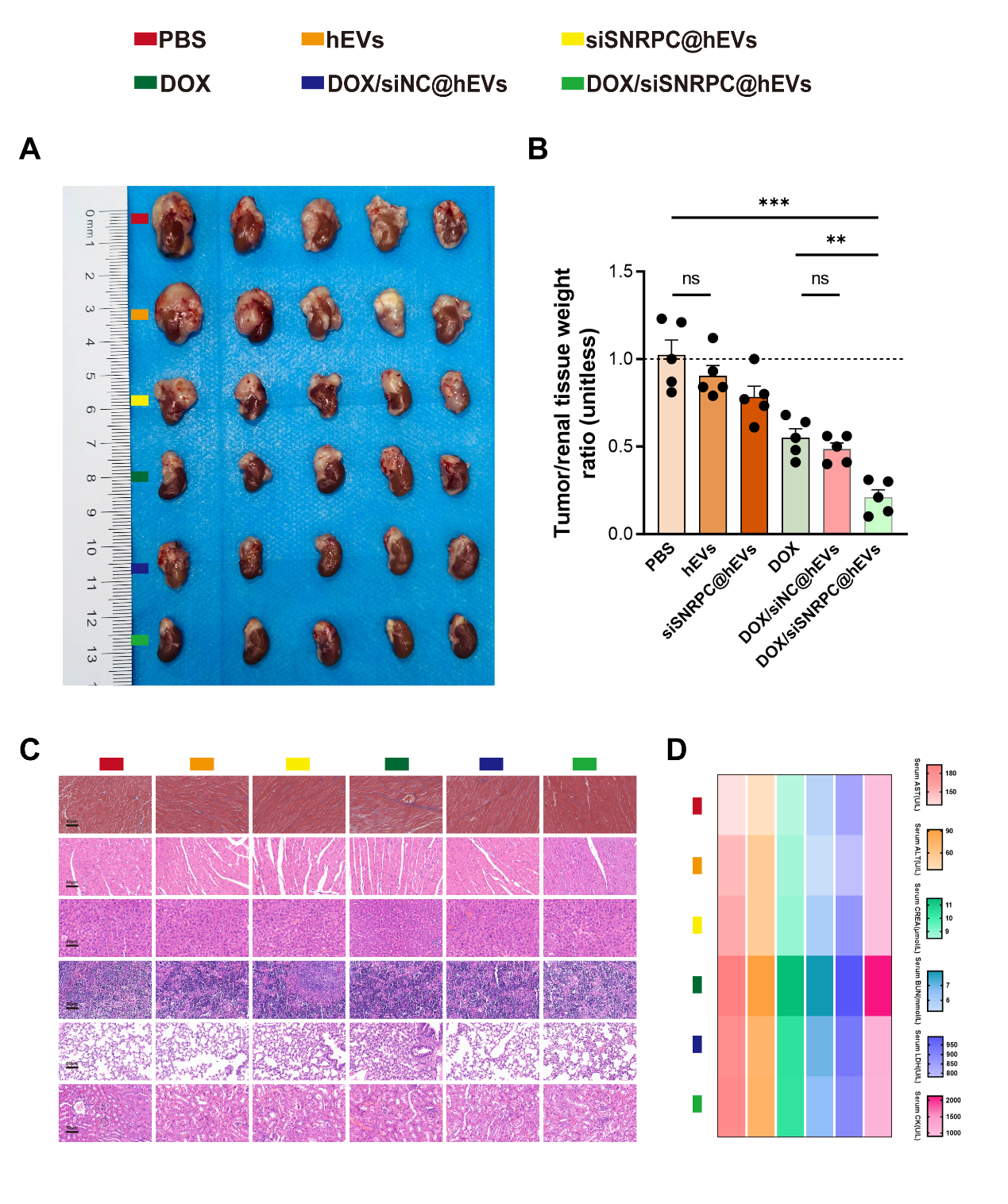

Supplement: Supplementary file 4 — Supplementary Material 4: Fig. S1. Screening of Potential Chemoresistance-Related Genes Associated with Macrophage Polarization in WT via Immunofluorescence Staining and Flow Cytometry. A. Immunofluorescence staining was performed to detect the expression levels of PSMA4, PPIH, PFDN4 and CKS1B in clinical tumor tissues, adjacent non-tumor tissues, as well as chemoresistant and chemosensitive clinical tissues, respectively. B. Flow cytometry was used to detect the effects of SNRPC, PSMA4, PPIH, PFDN4 and CKS1B knockdown on M2 polarization of macrophages under co-culture conditions, respectively. Fig. S2. Predicted structural model of p-P65 binding to the CXCL17 promoter using AlphaFold3. A. Predicted binding sites of p-P65 to the CXCL17 promoter using the JASPER database. B. The spatial structure of the potential binding sites between p-P65 and the CXCL17 promoter predicted by AlphaFold3 was visualized using PyMOL. C. Relative CXCL17 mRNA levels in control, TNF-α, IKK16, SNRPC knockdown, and SNRPC overexpression stable cell lines. D. Secreted CXCL17 protein levels in cell supernatants from the indicated groups. Fig. S3. Migrasome detection assays. A. Flow Chart of the Research Procedure. B. Expression changes of migrasome-related genes TSPAN4, TSPAN7, and TSPAN9 after SNRPC knockdown. C. WGA staining showing migrasome formation in WiT49/WT-CLS1 cells with SNRPC overexpression (oe_SNRPC) or silencing. D. ELISA analysis of CXCL17 concentration in cell supernatants after TSPAN4 knockdown in WiT49/WT-CLS1 cells. E. Flow cytometry analysis of CD68⁺CD206⁺ macrophage proportion in the Transwell co-culture system, with TSPAN4-silenced WiT49/WT-CLS1 cells in the upper chamber and PMA-treated THP-1 cells in the lower chamber. Fig. S4. Effects of DOX/siSNRPC@hEVs on CXCL17 Secretion and M2-TAM Polarization in Tumor Tissues. A. ELISA was performed to detect the content of CXCL17 in mouse tumor tissues; B. Immunofluorescence staining was conducted to observe the polarization of M [file 13046_2026_3680_MOESM4_ESM.docx]
